# Type I interferon signaling in malignant blasts contributes to treatment efficacy in AML patients

**DOI:** 10.1038/s41419-023-05728-w

**Published:** 2023-03-24

**Authors:** Peter Holicek, Iva Truxova, Jana Rakova, Cyril Salek, Michal Hensler, Marek Kovar, Milan Reinis, Romana Mikyskova, Josef Pasulka, Sarka Vosahlikova, Hana Remesova, Iva Valentova, Daniel Lysak, Monika Holubova, Petr Kaspar, Jan Prochazka, Lenka Kasikova, Radek Spisek, Lorenzo Galluzzi, Jitka Fucikova

**Affiliations:** 1 Sotio Biotech, Prague, Czech Republic; 2 Department of Immunology, Charles University, 2nd Faculty of Medicine and University Hospital Motol, Prague, Czech Republic; 3 Institute of Hematology and Blood Transfusion, Prague, Czech Republic; 4 Institute of Clinical and Experimental Hematology, 1st Faculty of Medicine, Charles University, Prague, Czech Republic; 5 Laboratory of Tumor Immunology, Institute of Microbiology of the Czech Academy of Sciences, Prague, Czech Republic; 6 Laboratory of Immunological and Tumour Models, Institute of Molecular Genetics of the Czech Academy of Sciences, Prague, Czech Republic; 7 Department of Hematology and Oncology, Faculty Hospital in Pilsen, Pilsen, Czech Republic; 8 Biomedical Center, Medical Faculty in Pilsen, Charles University, Pilsen, Czech Republic; 9 Czech Centre for Phenogenomics, Institute of Molecular Genetics of the Czech Academy of Sciences, Prague, Czech Republic; 10 Department of Radiation Oncology, Weill Cornell Medical College, New York, NY USA; 11 Sandra and Edward Meyer Cancer Center, New York, NY USA; 12 Caryl and Israel Englander Institute for Precision Medicine, New York, NY USA

**Keywords:** Cancer microenvironment, Immune cell death, Immunization, Prognostic markers

## Abstract

While type I interferon (IFN) is best known for its key role against viral infection, accumulating preclinical and clinical data indicate that robust type I IFN production in the tumor microenvironment promotes cancer immunosurveillance and contributes to the efficacy of various antineoplastic agents, notably immunogenic cell death inducers. Here, we report that malignant blasts from patients with acute myeloid leukemia (AML) release type I IFN via a Toll-like receptor 3 (TLR3)-dependent mechanism that is not driven by treatment. While in these patients the ability of type I IFN to stimulate anticancer immune responses was abolished by immunosuppressive mechanisms elicited by malignant blasts, type I IFN turned out to exert direct cytostatic, cytotoxic and chemosensitizing activity in primary AML blasts, leukemic stem cells from AML patients and AML xenograft models. Finally, a genetic signature of type I IFN signaling was found to have independent prognostic value on relapse-free survival and overall survival in a cohort of 132 AML patients. These findings delineate a clinically relevant, therapeutically actionable and prognostically informative mechanism through which type I IFN mediates beneficial effects in patients with AML.

## Introduction

Type I interferon (IFN) was initially discovered as a key component of a first-line defense system that increases the resistance of mammalian cells to viral pathogens [1–3]. An abundant preclinical and clinical literature emerging over the past decade demonstrate that type I IFN also supports natural and therapy-driven cancer immunosurveillance [4, 5]. In humans, type I IFN is a family of 17 proteins, encompassing 13 isoforms of interferon alpha (IFNA, best known as IFN-α), interferon beta 1 (IFNB1, best known as IFN-β), interferon epsilon (IFNE, best known as IFN-ε), interferon kappa (IFNK, best known as IFN-κ) and interferon omega 1 (IFNW1, best known as IFN-ω) [6, 7]. Type I IFN synthesis and secretion is generally elicited by the activation of pattern recognition receptors (PRRs), which are evolutionary ancient sensors for microbial products and endogenous danger signals commonly known as damage-associated molecular patterns (DAMPs) [8–10].

Type I IFN signals through an ubiquitous heterodimeric receptor consisting of interferon alpha and beta receptor subunit 1 (IFNAR1) and IFNAR2, culminating with the coordinated transactivation of numerous IFN-stimulated genes (ISGs) [3, 7, 11]. ISG synthesis affects a variety of biological processes ranging from resistance against viral infection to angiogenesis and immune activation [3, 7]. With specific respect to cancer, accumulating evidence indicates that while indolent and chronic type I IFN responses may be detrimental and support immunoevasion and tumor progression [12–16], robust and acute type I IFN signaling promotes tumor-targeting immunity by boosting both the priming and effector phase of the response [6, 15–18].

Supporting this notion, the intratumoral abundance of type I IFN or ISGs has been positively correlated with tumor infiltration by effector immune cells, signs of active anticancer immunity and favorable disease outcome in a variety of solid tumors [19, 20]. Conversely, single nucleotide polymorphisms negatively affecting the function of type I IFN-eliciting PRRs (as well as reduced levels of said PRRs or their signal transducers) have been consistently linked with immunosuppression in the tumor microenvironment (TME) and poor disease outcome [21–23]. Moreover, accumulating preclinical and clinical evidence indicates that the efficacy of numerous clinically employed anticancer regimens including conventional chemotherapeutics [4, 24, 25], radiation therapy [17, 26, 27], targeted anticancer agents [5, 28], immunotherapy [29] and non-viral oncolytic agents [30–32] rely on intact type I IFN signaling. Of note, unmodified or pegylated variants of human recombinant IFN-α2a or IFN-α2b have been approved by the US Food and Drug Administration (FDA) and other regulatory agencies worldwide for use in patients with various neoplasms, including chronic myelogenous leukemia (CML) [33]. An abundant literature suggests that type I IFN may also be beneficial for (at least some subsets of) patients with acute myeloid leukemia (AML) [34, 35].

Here, we report that peripheral blood mononuclear cells (PBMCs) from patients with AML express higher type I IFN levels that their counterparts from healthy donors, with malignant blasts being the major type I IFN source. While in AML patients with high type I IFN signaling active anticancer immunity is suppressed by malignant cells, type I IFN appears to mediate direct cytostatic, cytotoxic and chemosensitizing effects in multiple models of AML. In line with this notion, genetic signatures of type I IFN signaling were linked with improved relapse-free survival (RFS) and overall survival (OS) in a large cohort of patients with AML (*n* = 132), delineating a clinically actionable pathway with therapeutic and prognostic applications.

## Materials and methods

### Patients and samples

One hundred and thirty-two patients diagnosed with acute myeloid leukemia (AML) and treated at the Institute of Hematology and Blood Transfusion in Prague between March 2008 and April 2019 were enrolled in retrospective Study Cohort 1 (Table 1). One hundred and fifty-two patients diagnosed with AML from The Cancer Genome Atlas (TCGA) public were used as validation Study Cohort 2. Nine patients diagnosed with AML and treated at the Institute of Hematology and Blood Transfusion in Prague between April 2019 and May 2021 along with seven patients diagnosed with AML and treated at Department of Hemato-oncology of Faculty Hospital Pilsen between April 2019 and December 2021 were enrolled in prospective Study Cohort 3 (Supplemental Table 1). Informed consent was obtained according to the Declaration of Helsinki, and the study was approved by the local ethics committee. Peripheral blood samples obtained before the onset of chemotherapy were drawn into the 9 mL EDTA-coated tubes. Serum was collected and stored at −80 °C. Peripheral blood mononuclear cells (PBMCs) were isolated by Ficoll-Paque PLUS (GE Healthcare) gradient centrifugation and used for immediate downstream cell analyses or cryopreserved using CryoStor^®^ CS10 (StemCell Technologies) in liquid nitrogen for later use. An EasySep kit (StemCell Technologies) was employed to separate or deplete CD33^+^ malignant blasts from PBMCs.

**Table 1:** Main clinical and biological characteristics of AML patients from Study Cohort 1.

Variable	Study Cohort 1 *n* = 132
Age at diagnosis	
<50 years	60 (45%)
≥50 years	72 (55%)
Median (years)	52
Range (years)	19–68
Sex	
Male	71 (54%)
Female	61 (46%)
Peripheral-blood white cell count	
< 30.000/mm^3^	66 (50%)
≥ 30.000/mm^3^	66 (50%)
Median (109 cells/l)	30.1
Range (109 cells/l)	0.9–414.12
Blasts peripheral blood	
Median (%)	28
Range (%)	0–99
Blasts bone marrow	
Median (%)	56
Range (%)	2 to 96
De novo AML	113 (86%)
Secondary AML	
MDS/MPN, *n*	4 (3%)
Therapy related, *n*	10 (8%)
Not specified, *n*	9 (7%)
FAB classification	
M0	4 (3%)
M1	27 (21%)
M2	29 (22%)
M4	44 (33%)
M5	23 (17%)
M6	5 (4%)
Cytogenetic profile	
Favorable	14 (10%)
Intermediate	86 (65%)
Adverse	20 (16%)
Missing data	12 (9%)
Molecular characteristics	
DNMT3A	39
FLT3-ITD	38
KMT2A	2
GATA2	3
RUNX1::RUNX1T1	8
CBFB::MYH11	5
NPM1	41
CEBPA	8
Induction chemotherapy	
Daunorubicin + Ara-C (3 + 7)	87 (66%)
Idarubicin + Ara-C (3 + 7)	42 (32%)
BIDFA	1 (>1%)
FLA-IDA	1 (>1%)
HAM	1 (>1%)
Complete remission rate	112 (85%)
Consolidation	
Chemotherapy only	58 (44%)
HSCT	74 (56%)

*AML* acute myeloid leukemia, *Ara-C* cytarabine, *BIDFA* twice daily fludarabine and cytarabine, *CBFβ::MYH11* core-binding factor subunit beta - myosin heavy chain 11 fusion protein, *CEBPA,* CCAAT enhancer binding protein alpha, *DNMT3A* DNA methyltransferase 3 alpha, *FAB* French-American-British, *FLA-IDA* fludarabine-idarubicin, *FLT3-ITD* fms related receptor tyrosine kinase 3 - internal tandem duplication, *GATA2* GATA Binding Protein 2, *HAM* high-dose cytosine arabinoside and mitoxantrone, *HSCT* hematopoietic stem cell transplantation, *KMT2A* lysine methyltransferase 2A, *MDS* myelodysplastic syndrome, *MPS* myeloproliferative neoplasm, *NPM1* nucleophosmin 1, *RUNX1::RUNX1T1* RUNX family transcription factor 1 - RUNX1 partner transcriptional co-repressor 1 fusion protein.

### Cell lines and in vitro assays

Human AML KASUMI-1, MOLM-13 and MV4–11 cells as well as human CML K562 cells were a kind gift from Júlia Starková (CLIP - Childhood Leukaemia Investigation Prague). Further details about cell culture are provided in Suppl. Material and Methods. Polyinosinic:polycytidylic acid (poly(I:C), from InvivoGen), CpG oligodeoxynucleotides (ODN2216, from Invivogen) and TLR3/dsRNA specific complex inhibitor (TLRi, from Merck) were added to culture media to final concentrations of 50 µg/mL, 1,5 µM and 25 µM, respectively, for the indicated time. Recombinant human interferon-alpha (rIFN-α, from Bio-Techne) and recombinant human interferon-beta (rIFN-β, from PeproTech) were used at a final concentration of 500 pg/mL. AML cells were incubated with both rIFN-α and rIFN-β for 7 days, AML primary blasts for 3 days, leukemic stem cells (LSCs) for 5 days and PBMCs/CD33^+^ cell depleted PBMCs for 24 h at 37 °C in 5% CO_2_ humidified atmosphere before the analysis of phenotype, function and apoptosis by flow cytometry. Human rIFN-α and rIFN-β were re-administered into fresh media every 48 h during the incubation period. Chemotherapeutic drugs commonly used for treatment of AML including daunorubicin (DNR; KASUMI-1: 200 nM, MOLM-13: 150 nM, MV4–11: 400 nM, CD33^+^ blasts: 500 nM, LSCs: 125 nM) (Sigma-Aldrich) and cytarabine (Ara-C; KASUMI-1: 500 nM, MOLM-13: 1 nM, MV4–11: 500 nM, CD33^+^: 125 nM, LSCs: 125 nM) (Sigma-Aldrich) were used for the induction of apoptosis over a 24 h course. CD33^+^ malignant cells were incubated with an IFNAR1-blocking antibody (αIFNAR, from ThermoFisher - MMHAR-2) or isotype control (Iso, from ThermoFisher - PPV-04) at a final concentration of 8 µg/mL for 24 h, and subsequently at a concentration of 2 µg/mL for 72 h, without culture further replacements in culture medium.

### Quantitative real-time PCR (RT-qPCR)

Gene expression levels were evaluated on a CFX 96™ Real-Time System (Bio-Rad) using custom-designed primers and probes (500 nM and 200 nM final concentration, respectively) (Generi Biotech) (Supplemental Table 2) and KAPA PROBE Fast Master Mix (Kapa Biosystems). Relative gene expression levels were calculated using the ΔΔCt method and were normalized to the expression level of reference gene *SURF1* selected by Normfinder (GenEx software, MultiD Analyses). Samples below the detection limit were assigned a relative expression value of 0.

### Flow cytometry

PBMCs, malignant blasts, LSCs and cultured tumor cells were stained with multiple panels of fluorescent primary antibodies, appropriate isotype controls and fixable viability dyes to exclude live/dead cells (Supplemental Table 3). For the in vitro assessment of apoptosis, cells were stained with Annexin V for 20 min at 4 °C and 4′,6-diamidin-2-fenylindol (DAPI) (0.1 µg/mL) was added to cell suspension shortly prior to sample acquisition. Flow cytometry data were acquired on an LSRFortessa Analyzer (BD Biosciences) and analyzed with FlowJo v10.0 (Tree Star, Inc.).

### Degranulation and IFN-γ production after in vitro stimulation

To assess natural killer (NK) cell and T cell function in whole PBMCs or CD33^+^ cell-depleted PBMCs from AML patients, PBMCs were stimulated with 50 ng/mL phorbol 12-myristate 13-acetate (PMA, from Sigma Aldrich) plus 1 μg/mL ionomycin or with K562 cells at an effector:target ratio 10:1 in the presence of anti-CD107a antibody (eBioscience) for 1 h, followed by 3 h incubation with brefeldin A (BioLegend). Cells were then washed in PBS, stained with antibodies specific for surface markers (Supplemental Table 3), fixed in fixation/permeabilization buffer for 15 min (eBioscience), washed with permeabilization buffer and then stained with antibodies targeting a panel of intracellular markers (Supplemental Table 3).

### Leukemic stem cells (LSCs)

LSCs were isolated from the PBMCs of AML patients as follows. Thawed cell suspensions were depleted of dead cells by magnetic separation using the Dead Cell Removal Kit (Miltenyi Biotech) and subsequently CD34^+^ cells were isolated using CD34 MicroBead Kit UltraPure (Miltenyi Biotech), according to the manufacturer’s protocols. LSCs were determined as CD45^dim^, Lin^−^ (CD3^−^CD14^−^CD16^−^CD19^−^CD20^−^CD56^−^), CD34^+^, CD38^+/−^ and CD123^+/dim^ colony forming cells, as determined by flow cytometry and colony-forming assay (Supplemental Fig. 1A–C).

### *IFNAR2* deletion

KASUMI-1^*IFNAR2-/-*^ cells were prepared by the CRISPR/Cas9 technology (Supplemental Fig. 2 and Supplemental Table 4). Briefly, KASUMI-1 cells were electroporated with a mixture of *IFNAR2*-specific gRNAs incorporated in a pSpCas9(BB)−2A-GFP (PX458) expression vector for dual expression of Cas9 and gRNAs. Two days after electroporation, GFP^+^ cells were single sorted into 96-well plates coated with NSG mice bone marrow cells and expanded. Clone selection was performed based on (i) RT-qPCR specific for the *IFNAR2* (ii), detection of IFNAR2 by flow cytometry, and (iii) sensitivity to daunorubicin.

### Statistical analysis

Statistical analyses were performed on GraphPad Prism 8, R v. 3.6.1 and R Studio. 3.6.0. The distributions of data sets were tested by Shapiro-Wilk Test, determining the use of the parametric or non-parametric tests for subsequent analyses. Paired and unpaired Student’s t tests, as well as Wilcoxon and Mann-Whitney tests were used to assess differences between two groups. Differences among three or more groups were calculated using one-way ANOVA or Kruskal-Wallis tests corrected for multiple comparison by Holm-Sidak’s or Dunn’s tests. Pearson or Spearman correlations were used to evaluate the degree of the relationship between variables. Survival analyses were assessed for statistical significance with Log-rank tests. Univariate and multivariate Cox proportional hazard analysis were performed to assess the association of clinicopathological or immunological parameters with RFS and OS. Selected variables used in multivariate Cox regression hazard analysis exhibited no mutual collinearities, calculated by linear and logistic regressions and variance inflation factor (VIF). *p* values are reported and were considered not significant when >0.05.

## Results

### Cell-autonomous type I interferon (IFN) responses in acute myeloid leukemia (AML) patients

**Fig. 1:**
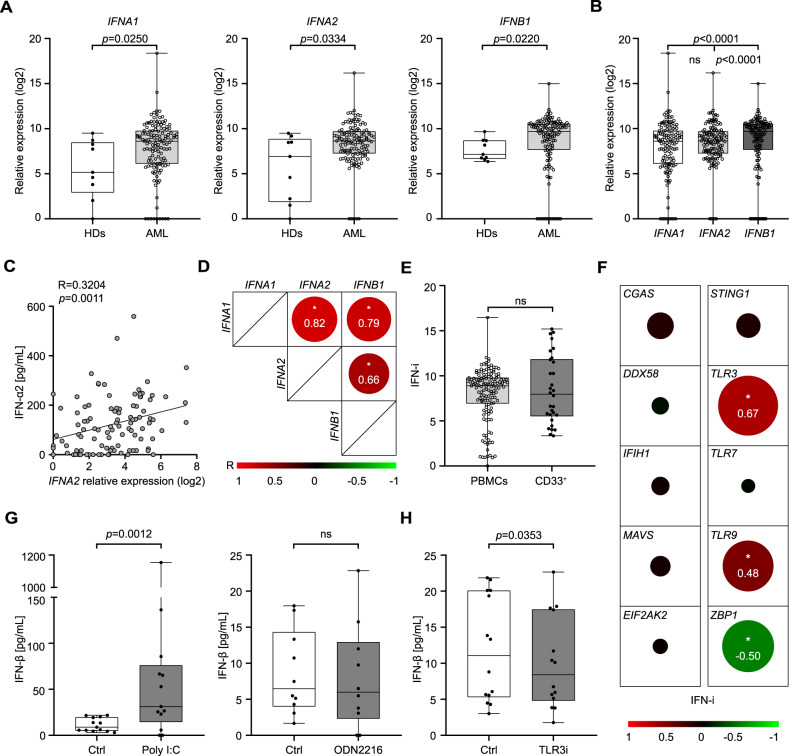
TLR3 drives type I IFN secretion from AML blasts. **A**, **B** Relative expression levels of *IFNA1*, *IFNA2*, and *IFNB1* in peripheral blood mononuclear cells (PBMCs) from 9 healthy donors (HDs) and 132 AML patients (Study Cohort 1) prior to induction chemotherapy, as determined by RT-qPCR. Data are presented as median, quartiles and extremes plus individual data points. *p* values are reported (Mann-Whitney test). **C** Correlation between IFNA2 serum levels and *IFNA2* expression in 101 AML patients (Study Cohort 1), as determined by Luminex and RT-qPCR, respectively. Spearman correlation coefficient (R) and associated *p* value are reported. **D** Correlation matrix for *IFNA1*, *IFNA2* and *IFNB1* expression in 132 AML patients from Study Cohort 1. Spearman correlation coefficient (R) is reported; *p < 0.0001. **E** Relative expression abundance of type I IFN index (IFN-i) in whole PBMCs (*n* = 132) versus isolated CD33^+^ malignant blasts (*n* = 30) from AML patients. Data are reported are presented as median, quartiles and extremes plus individual data points. ns, not significant (Mann-Whitney test). **F** Correlation matrix between IFN-i and relative expression levels of *CGAS, DDX58, IFIH1, MAVS, EIF2AK2, STING1, TLR3, TLR7, TLR9* and *ZBP1* in CD33^+^ leukemic blasts isolated from 30 AML patients (Study Cohort 1). Significant Spearman correlation coefficients (R) are reported; *p < 0.05. **G**, **H** IFN-β production by CD33^+^ blasts 24 h after optional treatment with polyI:C (*n* = 10), ODN2216 (CpG) (*n* = 10) **G** or a TLR3 inhibitor (TLR3i) (*n* = 10) **H**, as determined by ELISA. Data are presented as median, quartiles and extremes plus individual data points. Significant *p* values are reported; ns, not significant (Mann-Whitney test).

To elucidate the impact of type I IFN in AML immunosurveillance, we determined the expression levels of *IFNA1*, *IFNA2* and *IFNB1* in peripheral blood mononuclear cells (PBMCs) from 132 AML patients (Study Cohort 1; Table 1) by RT-qPCR. Chemotherapy-naïve AML patients exhibited increased levels of *IFNA1*, *IFNA2* and *IFNB1* as compared to healthy donors (HDs) (Fig. 1A). In this setting, we observed rather heterogeneous expression of type I IFN-encoding genes, *IFNB1* being the most abundantly expressed (Fig. 1B; Supplemental Fig. 3A). To validate these findings with an independent technology, we employed multiplex bead assays to quantify IFN-α2 levels in the serum of patients form Study Cohort 1 (Table 1). In line with RT-qPCR findings, the serum levels of IFN-α2 were heterogenous across patients, ranging from undetectable to 559 pg/mL (Fig. 1C). Importantly, we observed a statistically significant correlation between IFN-α2 serum levels and *IFNA2* expression in PBMCs from the AML patients of Study Cohort 1 for which both data points were available (R = 0.3204, *p* = 0.0011, *n* = 100) (Fig. 1C). As *IFNA1, IFNA2* and *IFNB1* expression exhibited considerable mutual correlation (Fig. 1D), we defined a type I IFN index (IFN-i) as the geometrical average of individual expression values for *IFNA1*, *IFNA2* and *IFNB1* to use in subsequent analyses.

Considering that leukemic blasts make up majority of blood cells in AML patients, we moved onto assessing the cellular source of type I IFN by testing *IFNA1, IFNA2*, and *IFNB1* expression levels in isolated CD33^+^ leukemic blasts *versus* whole PBMCs (including CD33^+^ blasts). We found that type I IFN expression was comparable in leukemic blasts and whole PBMCs (Fig. 1E, Supplemental Fig. 3A), pointing to the former as the major type I IFN producers in this context. Further corroborating this possibility, we observed a correlation between *IFNB1* levels in isolated CD33^+^ leukemic blasts and whole PBMCs from 22 AML patients (R = 0.4243, *p* = 0.0491) (Supplemental Fig. 3B). These findings indicate that malignant blasts from AML patients produce type I IFN prior to induction chemotherapy.

### TLR3 drives type I IFN secretion from AML blasts

To delineate the molecular pathway responsible for type I IFN production in AML patients, we analyzed the expression of genes coding for common DNA/RNA sensors that are known to elicit type I IFN signaling [8, 16], including cyclic GMP–AMP synthase (CGAS), DExD/H-Box Helicase 58 (DDX58; best known as RIG-I), interferon induced with helicase C domain 1 (IFIH1; best known as MDA5), mitochondrial antiviral signaling protein (MAVS), eukaryotic translation initiation factor 2-alpha kinase 2 (EIF2AK2; best known as PKR), stimulator of interferon response cGAMP interactor 1 (STING1), TLR3, TLR7, TLR9 and Z-DNA binding protein 1 (ZBP1) on CD33^+^ malignant blasts isolated from 30 AML patients of Study Cohort 1. We observed a significant positive correlation between IFN-i and the expression levels of *TLR3* (R = 0.67; *p* < 0.0001) and *TLR9* (R = 0.48; *p* = 0.0021), as well as a negative correlation between IFN-i and *ZBP1* levels (R = −0.50; *p* = 0.0274) (Fig. 1F, Supplemental Fig. 3C). These findings were confirmed on the entire Study Cohort 1 using *TLR3* (R = 0.5610; *p* < 0.0001) and *TLR9* (R = 0.3238; *p* = 0.0002) expression levels in PBMCs (Supplemental Fig. 3D). To corroborate our data in an independent cohort of AML patients, we retrieved normalized *TLR3* expression levels of 152 AML patients from The Cancer Genome Atlas (TCGA) public database (Study Cohort 2), confirming a significant correlation between *IFNB1* and *TLR3* levels (R = 0.3406; *p* < 0.0001) (Supplemental Fig. 3E).

To estimate the functional impact of TLR3 and TLR9 signaling on type I IFN production by leukemic blasts, we harnessed ELISA to measure IFN-β synthesis by isolated CD33^+^ blasts after TLR3 *versus* TLR9 stimulation. We observed a significant increase in IFN-β levels in response to the TLR3 agonist polyinosinic:polycytidylic acid [poly(I:C)], but not to the TLR9 agonist ODN2216 (Fig. 1G). Moreover, IFN-β secretion by otherwise unstimulated CD33^+^ malignant blasts was significantly reduced upon inhibition of TLR3 with a TLR3/dsRNA-specific complex inhibitor (TLR3i) (Fig. 1H).

We next aimed at determining the signal transduction pathways elicited by TLR3 in support of type I IFN secretion in blasts from AML patients. To this aim, we compared the phosphorylation status of the TLR3 signal transducer TANK binding kinase 1 (TBK1) in 3 patients with AML exhibiting lower-than-median IFN-i (IFN-i ^Lo^) vs 3 patients with AML exhibiting higher-than-median IFN-i (IFN-i^Hi^) from Study Cohort 1 by immunoblot analyses (Supplemental Table 5). We found a trend for TBK1 to be hyperphosphorylated in IFN-i^Hi^ versus IFN-i ^Lo^ patients (Supplemental Fig. 4A, Supplemental Fig. 5), suggesting a preferential implication of this pathway. In further support of this notion, there was no difference in the abundance of a gene signature indicative of NF-κB signaling (*i.e*., *RELA, TRAF6, TAB1, RIPIL1, TNFRSF1B, TNFRSF1A, IL1R1, NFKBIA, MYD88, TNFAIP3, TRADD, TNF, NFKB1, FADD, CHUK, MAP3K1, MAP3K7, IKBKB, IKBKG, MAP3K14*) in 12 IFN-i^Lo^ vs 12 IFN-i^Hi^ patients from our study cohort 1 nor in 76 IFN-i^Lo^ vs 76 IFN-i^Hi^ patients from the TCGA dataset (Study Cohort 2; Supplemental Fig. 4B–D).

Taken together, these findings indicate that AML blasts produce type I IFN via TLR3-TBK1-IRF3 signaling.

### Type I IFN-driven immunostimulation is suppressed by malignant blasts

**Fig. 2:**
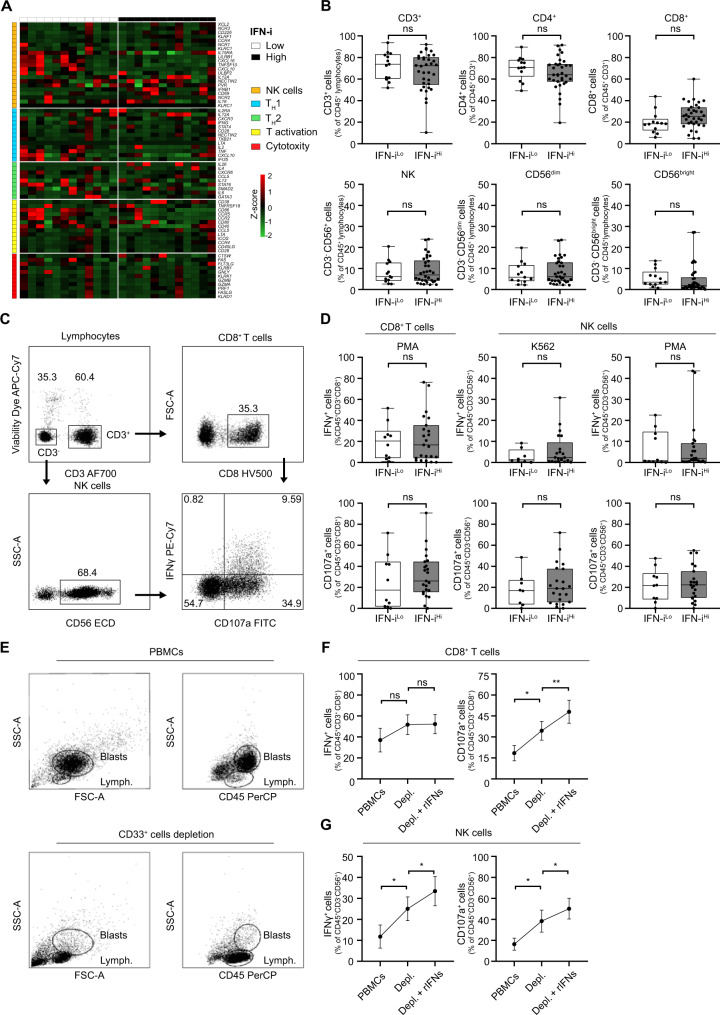
Type I IFN-driven immunostimulation is suppressed by malignant blasts. **A** Relative expression levels of selected genes associated with NK cells, T_H_1 and T_H_2 response, T cell activation and cytotoxicity in 12 IFN-i^Lo^ and 12 IFN-i^Hi^ AML patients from Study Cohort 1 as determined RNAseq (see Supplementary Table 4). **B** Percentage of circulating CD45^+^CD3^+^, CD45^+^CD3^+^CD4^+^, CD45^+^CD3^+^CD8^+^ T cells and CD45^+^CD3^−^CD56^+^, CD45^+^CD3^−^CD56^dim^ and CD45^+^CD3^−^CD56^bright^ NK cells in 13 IFN-i^Lo^ *versus* 34 IFN-i^Hi^ AML patients from Study Cohort 1 prior to induction chemotherapy, as determined by flow cytometry. Data are presented as median, quartiles and extremes plus individual data points. ns, not significant (Mann-Whitney test). **C** Gating strategy for IFN-γ^+^ and CD107a^+^ CD45^+^CD3^+^CD8^+^ T cells and CD45^+^CD3^−^CD56^+^ NK cells. The percentage of cells in each gate is reported. **D** Percentage of IFN-γ^+^ and CD107a^+^ CD45^+^CD3^+^CD8^+^ T cells and CD45^+^CD3^−^CD56^+^ NK cells upon stimulation with PMA plus ionomycin or K562 cells of peripheral blood mononuclear cells (PBMCs) from 10 IFN-i^Lo^ *versus* 23 IFN-i^Hi^ AML patients of Study Cohort 1, as determined by flow cytometry. Data are presented as median, quartiles and extremes plus individual data points. Significant *p* values are reported; ns, not significant (Mann-Whitney test). **E** Representative dot plots showing PBMC composition of one AML patient before and after depletion of CD33^+^ leukemic blasts. **F**, **G** Percentage of IFN-γ^+^ and CD107a^+^ CD8^+^ T cells and NK cells upon stimulation with PMA plus ionomycin of PBMCs optionally depleted of CD33^+^ blasts and optionally exposed to recombinant IFN-α plus IFN-β (rIFNs) from 7 AML patients of Study Cohort 1, as determined by flow cytometry. Data are reported as means ± SEM. *p < 0.05; **p < 0.01; ns, not significant (paired t-test).

To elucidate the immunostimulatory effects of type I IFN secreted by CD33^+^ malignant blasts, we first harnessed RNAseq and compared the gene expression profile of PBMCs from 12 IFN-i ^Lo^ *versus* 12 IFN-i^Hi^ patients from Study Cohort 1. While we identified a set of 433 differentially expressed genes (DEGs) (Supplemental Fig. 6A, Supplemental Table 6), pathway enrichment analyses failed to determine an association between upregulated DEGs and immune functions such as NK cell infiltration, T_H_1 polarization, T_H_2 polarization, T cell activation and cytotoxicity (Fig. 2A). These findings were confirmed in Study Cohort 2, suggesting a limited immunostimulatory effect from endogenous type I IFN in patients with AML (Supplemental Fig. 6B).

To corroborate these findings with an independent technology, we determined the frequency of circulating CD3^+^ lymphocytes, CD4^+^ T cells, CD8^+^ cytotoxic T cells, CD3^−^CD56^+^ NK cells as well as CD3^−^CD56^dim^ or CD3^−^CD56^bright^ NK cells, plus the phenotypic profile of dendritic cells (DCs) in IFN-i^Lo^ *versus* IFN-i^Hi^ patients from Study Cohort 1 using flow cytometry. In line with our previous observations, we failed to document any statistically significant difference in the abundance or functional profile of the aforementioned cell populations in this setting (Fig. 2B, Supplemental Fig. 6C). Along similar lines, both CD8^+^ T cells and NK cells from IFN-i^Lo^ patients were equally (and rather poorly) responsive to stimulation with phorbol 12-myristate 13-acetate (PMA) plus ionomycin (which non-specifically activate lymphoid cells) or K562 cells (an NK cell target) as their counterparts from IFN-i^Hi^ patients, both in terms of IFN-γ secretion and degranulation (as assessed by CD107a positivity) (Fig. 2C, D).

As malignant blasts are potent drivers of immunosuppression in AML [36], we next assessed the functional capacity of circulating CD8^+^ T cells and NK cells from 7 AML patients of Study Cohort 3 before and after depletion of CD33^+^ malignant blasts (Fig. 2E). We found that prior to CD33^+^ cell depletion, recombinant IFN-α and IFN-β (rIFNs) fails to improve the ability of CD8^+^ T cells and NK cells from AML patients to respond to PMA plus ionomycin with IFN-γ synthesis and degranulation (Supplemental Fig. 6D, E). Conversely, both CD8^+^ T cells and NK cells from AML patients were reinvigorated in their ability to respond to PMA plus ionomycin upon depletion of malignant blasts, and even more so depletion of malignant blasts combined with rIFNs administration (Fig. 2F, G), although the effect on IFN-γ synthesis was less pronounced on CD8^+^ T cells than on NK cells. Of note, the reinvigorated responsiveness of both CD8^+^ T cells and NK cells to PMA plus ionomycin upon CD33^+^ malignant blast depletion was compromised by the subsequent re-addition of isolated autologous CD33^+^ blasts (Supplemental Fig. 6F, G).

Taken together, these findings suggest that CD33^+^ malignant blasts actively impair baseline and type I IFN-stimulated CD8^+^ T cell and NK cell effector functions in AML patients.

### Recombinant type I IFN mediates direct cytostatic and cytotoxic activity on AML blasts and leukemic stem cells

**Fig. 3:**
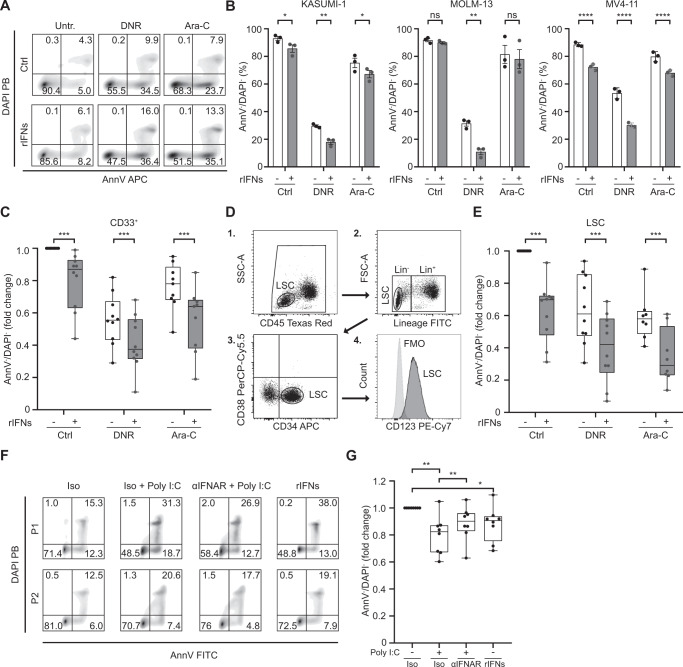
Recombinant type I IFN mediates direct cytostatic and cytotoxic activity on AML blasts and leukemic stem cells. **A**, **B** Representative dot plots **A** and percentages **B** of viable (Annexin V^−^/DAPI^−^) KASUMI-1, MOLM-13 and MV4–11 cells after daunorubicin (DNR) and cytarabine (Ara-C) 24 h treatment with optional recombinant IFN-α plus IFN-β (rIFNs) (500 pg/mL) 3 days pre-incubation, as determined by flow cytometry. Data are reported as means ± SD plus individual data points. *p < 0.05; **p < 0.01, ****p < 0.0001, ns: not significant (paired t-test). **C** Relative viability of CD33^+^ leukemic blasts from 10 AML patients (Study Cohort 3) after DNR or Ara-C treatment with optional 3 days pre-incubation with rIFNs (500 pg/mL). Data are reported as means, quartiles and extremes plus individual data points. ***p < 0.001 (paired t-test). **D** Gating strategy for determination of leukemic stem cells (LSCs) in AML patient PBMCs using flow cytometry. **E** Relative viability of LSCs isolated from 10 AML patients (Study Cohort 3) after DNR or Ara-C treatment with optional 3 days pre-incubation with rIFNs (500 pg/mL). Data are reported as means, quartiles and extremes plus individual data points. ***p < 0.001 (paired t-test). **F** Relative viability of CD33^+^ leukemic blasts from 8 AML patients (Study Cohort 3) 96 h upon exposure to polyI:C in the optional presence of an IFNAR1 blocking antibody, or rIFNs, Data are reported as means, quartiles and extremes plus individual data points. *p < 0.05, **p < 0.01 (paired t-test).

Type I IFN has previously been suggested to exert direct cytostatic and cytotoxic activity against neoplastic cells, including malignant leukemic blasts [6]. To validate these findings, we tested the effect of rIFNs on human KASUMI-1, MOLM-13 and MV4–11 AML cells, observing a considerable cytostatic activity using a [^3^H]-thymidine incorporation assay (Supplemental Fig. 7A). Similarly, rIFNs exerted some degree of cytotoxicity against human KASUMI-1, MOLM-13 and MV4–11 AML cells, as determined by flow cytometry (Fig. 3A, B), which could generally build on the effects of standard of care (SOC) chemotherapeutics including daunorubicin (DNR) and/or cytarabine (Ara-C) (Fig. 3B). We next determined the cytotoxic activity of rIFNs on primary blasts from AML patients, also observing direct cytotoxicity from rIFNs and additive effects when rIFNs were delivered along with DNR or Ara-C (Fig. 3C). As disease outcome in AML patients is often determined by the resistance of leukemic stem cells (LSCs) to SOC therapy [37], we next analyzed the impact of rIFNs on LSC viability (Fig. 3D). Importantly, rIFNs also mediated direct cytotoxicity on LSCs, as determined by flow cytometry, an effect was even more pronounced in the presence of DNR or Ara-C (Fig. 3E).

To directly estimate the cytotoxicity of TLR3 signaling in primary AML blasts and obtain insights in the underlying mechanisms, we nest stimulated CD33^+^ cells isolated from 8 AML patients of Study Cohort 1 with the TLR3 agonist polyI:C in the optional presence of an IFNAR1-blocking antibody. We observed a loss in cellular viability driven by polyI:C comparable to that observed upon exposure of primary AML blasts to rIFNs (Fig. 3C), which could be at least partially counteracted by IFNAR1 blockage (Fig. 3F, G).

Altogether, these findings document the cytostatic and cytotoxic effect of type I IFN (employed at concentrations that are detected in the circulation of AML patients) as secreted downstream of TLR3 activation on both AML malignant blasts and LSCs.

### Chemosensitizing effects of type I IFN in human AML xenografts

**Fig. 4:**
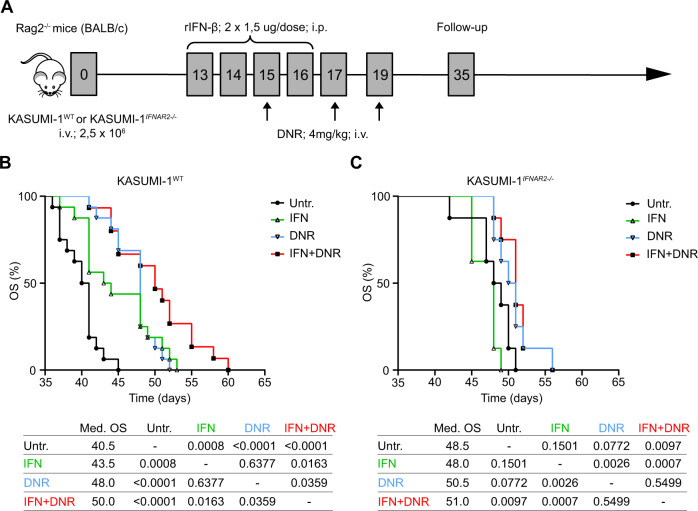
Chemosensitizing effects of type I IFN in human AML xenografts. **A** Experimental study design of an AML xenograft model using human WT or *IFNAR2*^*-/-*^ KASUMI-1 cells in *Rag2*^*-/-*^ mice. **B** Overall survival (OS) of *Rag2*^*-/-*^ mice xenografted with WT KASUMI-1 cells and optionally treated with human rIFN-β (IFN), daunorubicin (DNR) or IFN + DNR. **C** OS of *Rag2*^*-/-*^ mice xenografted with *IFNAR2*^*-/-*^ KASUMI-1 cells and optionally treated with IFN, DNR or IFN + DNR. Survival curves were estimated by the Kaplan-Meier method, and differences between groups were evaluated using log-rank test. Median OS (days) and *p* values are reported.

To examine the impact of exogenous type I IFN on the efficacy of SOC chemotherapy, we generated AML xenografts by intravenously injecting *Rag2*^*-/-*^ mice (which lack B and T cells) with 2.5 × 10^6^ human wild-type (WT) KASUMI-1 AML cells (Fig. 4A). Human rIFN-β was optionally administered over 4 consecutive days and 2 days prior to chemotherapy initiation in an attempt to mimic the baseline status of AML patients (Fig. 4A). In line with our in vitro findings, both type I IFN (median OS:43.5 days; *p* = 0.0008) and DNR (median OS:48.0 days; *p* < 0.0001) extended the OS of Rag2^*-/-*^ mice bearing WT KASUMI-1 cells, as compared to untreated mice (median OS: 40.5 days), an effect that was magnified when type I IFN and DNR were combined (median OS:50.0 days; *p* = 0.0359 *versus* DNR; *p* = 0.0163 *versus* type I IFN) (Fig. 4B). To rule out potential interferences emerging from any cross-reactivity between human rIFN-β and mouse type I IFN receptors, we repeated the same experiments with *IFNAR2*^*-/-*^ KASUMI-1 cells. Importantly, in the absence of IFNAR2, KASUMI-1 xenografts became irresponsive to human rIFN-β (median OS:48.0 days; *p* = 0.1501) and also poorly responsive to DNR (median OS: 50.5 days; p = 0.0772) (Fig. 4C). Along these lines, combining DNR with human rIFN-β offered no survival advantages to mice bearing *IFNAR2*^*-/-*^ KASUMI-1 xenografts as compared to DNR alone (median OS: 51.0 days; *p* = 0.5499) (Fig. 4C).

These findings extend our previous observations to document a beneficial impact of cancer cell-autologous type I IFN signaling on AML treatment sensitivity.

### Type I IFN levels correlate with improved disease outcome in patients with AML

**Fig. 5:**
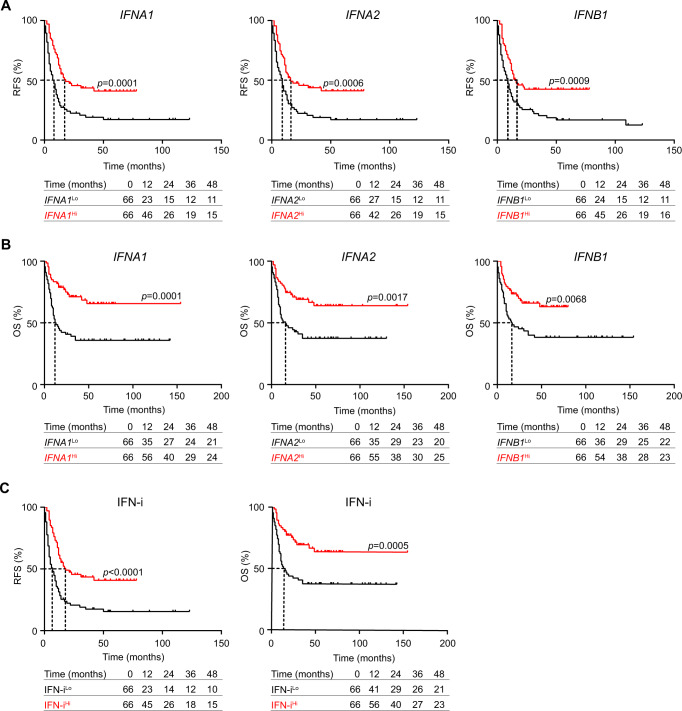
Type I IFN levels correlate with improved disease outcome in patients with AML. **A**–**C** Relapse-free (RFS) and overall survival (OS) of 132 AML patients from Study Cohort 1 upon median stratification based on *IFNA1*, *IFNA2*, *IFNB1* expression or type I IFN index (IFN-i). Survival curves were estimated by the Kaplan-Meier method, and differences between groups were evaluated using log-rank test. Number of patients at risk and *p* values are reported.

Inspired by our findings on the cytostatic and cytotoxic impact of type I IFN signaling on AML blasts and LSCs, we moved to determine the prognostic role of type I IFN genes in AML patients from Study Cohort 1 (*n* = 132) (Table 1), upon stratifying the entire patient cohort based median *IFNA1, IFNA2, IFNB1* expression level or median IFN-i values into IFN^Lo^ (*n* = 66) and IFN^Hi^ (*n* = 66) groups. IFN^Hi^ patients exhibited significantly improved RFS (*IFNA1*, HR: 0.41, *p* = 0.0001; *IFNA2*, HR: 0.50, *p* = 0.0006; *IFNB1*, HR: 0.49, *p* = 0.0009; IFN-i, HR:0.44; *p* < 0.0001) (Fig. 5A, C) and OS (*IFNA1*, HR: 0.37, *p* = 0.0001; *IFNA2*, HR: 0.44, *p* = 0.0017; *IFNB1*, HR: 0.50, *p* = 0.0068; IFN-i, HR: 0.40; *p* = 0.0005) (Fig. 5B, C) as compared to their IFN^Lo^ counterparts. On the contrary, the relative abundance of leukemic blasts in the peripheral blood or bone marrow failed to confer any prognostic information in this patient cohort (Supplemental Fig. 8A, B). These findings were confirmed by univariate Cox proportional hazard analyses (Table 2). Furthermore, multivariate Cox proportional hazard analysis identified IFN-i as a prognostic biomarker (RFS - HR: 0.91, CI95% 0.85–0.97, *p* = 0.003; OS - HR: 0.91, CI95% 0.84–0.99, *p* = 0.021) independent of clinical characteristics, including age, cytogenetic classification, hematopoietic transplantation, secondary AML and white blood cell (WBC) count (Table 3).

**Table 2:** Univariate Cox proportional hazard analysis.

	OS		RFS	
Variable	HR (95% CI)	*p* value	HR (95% CI)	*p* value
*IFNA1*	0.86 (0.80–0.92)	<0.0001	0.86 (0.81–0.92)	<0.0001
*IFNA2*	0.90 (0.82–0.98)	0.011	0.92 (0.86–0.99)	0.018
*IFNB1*	0.92 (0.87–0.98)	0.014	0.91 (0.86–0.96)	0.00056
IFN-i	0.88 (0.82–0.95)	0.0009	0.88 (0.82–0.93)	<0.0001
Age	1.10 (1.00–1.10)	<0.0001	1.00 (1.00–1.00)	0.12
Leukocytes	1.00 (1.00–1.00)	0.0069	1.00 (1.00–1.00)	0.01
PB - Blasts	1.00 (0.99–1.00)	0.39	1.00 (0.99–1.00)	0.74
BM - Blasts	1.00 (0.99–1.00)	0.59	1.00 (0.99–1.00)	0.59
MRD	1.00 (0.98–1.00)	0.60	0.99 (0.97–1.00)	0.65
HSCT				
	0.27 (0.16–0.40)	<0.0001	0.73 (0.48–1.10)	0.13
Cytogenetics favorable				
	0.62 (0.25–1.60)	0.3100	1.10 (0.63–2.00)	0.67
Cytogenetics intermediate				
	0.53 (0.31–0.92)	0.025	0.51 (0.32–0.80)	0.0032
Cytogenetics adverse				
	2.90 (1.60–5.20)	0.0006	2.70 (1.60–4.60)	<0.0001
Secondary AML				
	2.20 (1.20–3.80)	0.0064	1.20 (0.68–2.00)	0.59
*CEBPA*				
	0.65 (0.20–2.10)	0.46	0.52 (0.19–1.40)	0.21
*DNMT3A*				
	0.80 (0.45–1.40)	0.4500	1.20 (0.77–1.90)	0.40
*IDH1*				
	0.79 (0.28–2.20)	0.65	0.71 (0.31–1.60)	0.43
*IDH2*				
	1.10 (0.51–2.30)	0.85	0.91 (0.47–1.80)	0.79
*FLT3*-ITD				
	1.30 (0.75–2.20)	0.36	1.50 (0.98–2.40)	0.059
*NPM1*				
	1.00 (0.60–1.70)	0.96	0.99 (0.64–1.5)	0.95

*BM* bone marrow, *CEBPA* CCAAT/enhancer-binding protein alpha, *DNMT3A* DNA (cytosine-5)-methyltransferase 3A, *FLT3-ITD* fms related receptor tyrosine kinase 3 - internal tandem duplication, *HSCT* hematopoietic stem cell transplantation, *IFN-i* type I interferon index, *MRD* minimal residual disease, *NPM1* nucelophosmin 1, *PB* peripheral blood, *IDH1* isocitrate dehydrogenase (NADP( + )) 1, isocitrate dehydrogenase (NADP( + )) 2.

**Table 3:** Multivariate Cox proportional hazard analysis.

		OS		RFS	
Variable	Levels	HR (95% CI)	*p* value	HR (95% CI)	*p* value
Age		1.05 (1.02–1.08)	0.003	1.01 (0.99–1.03)	0.491
Cytogenetic risk	1				
	2	1.31 (0.50–3.46)	0.584	0.73 (0.39–1.36)	0.326
	3	5.17 (1.78–15.07)	0.003	1.92 (0.92–4.02)	0.084
HSCT	0				
	1	0.29 (0.15–0.54)	0.0001	0.82 (0.52–1.30)	0.408
IFN-i		0.91 (0.84–0.99)	0.021	0.91 (0.85–0.97)	0.003
Leukocytes		1.00 (1.00–1.01)	0.024	1.00 (1.00–1.00)	0.043
Secondary AML	0				
	1	1.66 (0.88–3.14)	0.120	1.14 (0.64–2.03)	0.665

*AML* acute myeloid leukemia, *HSCT* hematopoietic stem cell transplantation, *IFN-i* type I interferon index.

These findings suggest that type I IFN signaling may convey independent prognostic information in patients with AML.

## Discussion

Accumulating preclinical and clinical evidence indicates that, beyond a crucial role in curtailing viral infection, type I IFN produced by malignant cells and/or immune components of the TME contributes to clinically relevant cancer immunosurveillance in numerous oncological indications [38]. In line with this notion, type I IFN signaling supports the efficacy of various anticancer regimens including conventional chemotherapeutics [4, 24, 25], radiation therapy [17, 26, 27], targeted anticancer agents [5, 28], immunotherapy [29] and non-viral oncolytic agents [30–32].

Here, we harnessed two independent patient cohorts to define the immunobiology and prognostic relevance of type I IFN in AML. Specifically, we found that malignant blasts from AML patients release type I IFN via a TLR3-dependent mechanism that is not induced by treatment (Fig. 1). In this respect, our results extend previous findings documenting a crucial role of TLR3 signaling in type I IFN release by cancer cells [6, 24]. Such a signaling pathway most likely originates from endogenous RNA species released by a fraction of dying malignant blasts, as previously documented in other settings [24, 39].

Despite expectations, type I IFN release failed to correlate with signs of active antitumor immunity as mediated by T_H_1 CD4^+^ T cells, IFN-γ-producing CD8^+^ cells and NK cells (Fig. 2). Rather, the immunostimulatory function of type I IFN was impaired in AML patients by immunosuppressive mechanisms driven by malignant blasts (Fig. 2). These findings are in line with an ample preclinical and clinical literature demonstrating the potent immunosuppressive activity of leukemic blasts [40–42]. Potential mechanisms at play in this setting include (but may not be limited to): (i) the release of immunosuppressive cytokines like interleukin 10 (IL10) or tumor necrosis factor (TNF) downstream of TNF superfamily member 9 (TNFRSF9, best known as CD137) [40] or TNFSF18 signaling [41] and (ii); the direct inhibition of T cell and NK cell cytotoxic functions via CD200 [42]. Conversely, type I IFN at concentrations similar to those detected in AML patients mediated direct cytotoxic effects and cooperated with SOC chemotherapeutics (DNR, Ara-C) against leukemic blasts, in vitro (Fig. 3) and in vivo (Fig. 4). Similar results have previously been obtained with immunodeficient murine AML xenografts subjected to continuous delivery of type I IFN by an adenoviral vector [43].

Supporting the clinical relevance of our findings, type I IFN expression was independently associated with improved RFS and OS in patients with AML (Tables 2, 3). Consistent with this notion, type I IFN expression levels or type I IFN signaling signatures have previously been attributed prognostic value in patients with glioblastoma [44] and breast carcinoma with poor prognosis [24, 45]. That said, signatures of type I IFN signaling have also been linked to poor disease outcome in other cohorts of breast carcinoma patients [12, 13] and colorectal cancer patients [46, 47]. At least in part, such an apparent discrepancy may reflect the differential effect of potent/acute *versus* indolent/chronic type I IFN signaling and/or the overall immunological contexture of the TME [16].

Taken together, our findings corroborate previous preclinical studies documenting the antineoplastic activity of type I IFN in the AML setting [38, 48]. These findings inspired clinical studies investigating recombinant human IFN-α in different therapeutic settings, including (but not limited to): (i) induction (ii), salvage therapy for patients relapsing upon hematopoietic stem cell transplantation (HSCT), and (iii) post-remission consolidation therapy [34, 49] with objective clinical responses observed in all such settings. Our findings suggest that at least part of such a benefit may originate from direct cytotoxicity rather than from the activation of tumor-targeting immunity. Thus, we surmise that monitoring of type I IFN levels might improve the clinical management of AML patients.

## Supplementary information

Supplemental data

Checklist

## Data Availability

The data generated in this study are available upon request to the corresponding author.

## References

[1] Kroemer G, Galassi C, Zitvogel L, Galluzzi L (2022). Immunogenic cell stress and death. Nat Immunol.

[2] McNab F, Mayer-Barber K, Sher A, Wack A, O’Garra A (2015). Type I interferons in infectious disease. Nat Rev Immunol.

[3] Schoggins JW (2019). Interferon-stimulated genes: what do they all do?. Annu Rev Virol.

[4] Galluzzi L, Humeau J, Buque A, Zitvogel L, Kroemer G (2020). Immunostimulation with chemotherapy in the era of immune checkpoint inhibitors. Nat Rev Clin Oncol.

[5] Petroni G, Buque A, Zitvogel L, Kroemer G, Galluzzi L (2021). Immunomodulation by targeted anticancer agents. Cancer Cell.

[6] Borden EC (2019). Interferons alpha and beta in cancer: therapeutic opportunities from new insights. Nat Rev Drug Disco.

[7] Lukhele S, Boukhaled GM, Brooks DG (2019). Type I interferon signaling, regulation and gene stimulation in chronic virus infection. Semin Immunol.

[8] Vanpouille-Box C, Hoffmann JA, Galluzzi L (2019). Pharmacological modulation of nucleic acid sensors - therapeutic potential and persisting obstacles. Nat Rev Drug Disco.

[9] Gong T, Liu L, Jiang W, Zhou R (2020). DAMP-sensing receptors in sterile inflammation and inflammatory diseases. Nat Rev Immunol.

[10] Marchi S, Guilbaud E, Tait SWG, Yamazaki T, Galluzzi L (2022). Mitochondrial control of inflammation. Nat Rev Immunol.

[11] Saleiro D, Platanias LC (2019). Interferon signaling in cancer. Non-canonical pathways and control of intracellular immune checkpoints. Semin Immunol.

[12] Rodriguez-Ruiz ME, Buque A, Hensler M, Chen J, Bloy N, Petroni G (2019). Apoptotic caspases inhibit abscopal responses to radiation and identify a new prognostic biomarker for breast cancer patients. Oncoimmunology.

[13] Weichselbaum RR, Ishwaran H, Yoon T, Nuyten DS, Baker SW, Khodarev N (2008). An interferon-related gene signature for DNA damage resistance is a predictive marker for chemotherapy and radiation for breast cancer. Proc Natl Acad Sci USA.

[14] Erdal E, Haider S, Rehwinkel J, Harris AL, McHugh PJ (2017). A prosurvival DNA damage-induced cytoplasmic interferon response is mediated by end resection factors and is limited by Trex1. Genes Dev.

[15] Boukhaled GM, Harding S, Brooks DG (2021). Opposing roles of type I interferons in cancer immunity. Annu Rev Pathol.

[16] Vanpouille-Box C, Demaria S, Formenti SC, Galluzzi L (2018). Cytosolic DNA sensing in organismal tumor control. Cancer Cell.

[17] McLaughlin M, Patin EC, Pedersen M, Wilkins A, Dillon MT, Melcher AA (2020). Inflammatory microenvironment remodelling by tumour cells after radiotherapy. Nat Rev Cancer.

[18] Parker BS, Rautela J, Hertzog PJ (2016). Antitumour actions of interferons: implications for cancer therapy. Nat Rev Cancer.

[19] Cheon H, Borden EC, Stark GR (2014). Interferons and their stimulated genes in the tumor microenvironment. Semin Oncol.

[20] Linsley PS, Speake C, Whalen E, Chaussabel D (2014). Copy number loss of the interferon gene cluster in melanomas is linked to reduced T cell infiltrate and poor patient prognosis. PLoS ONE.

[21] Fucikova J, Moserova I, Urbanova L, Bezu L, Kepp O, Cremer I (2015). Prognostic and Predictive Value of DAMPs and DAMP-Associated Processes in Cancer. Front Immunol.

[22] Bidwell BN, Slaney CY, Withana NP, Forster S, Cao Y, Loi S (2012). Silencing of Irf7 pathways in breast cancer cells promotes bone metastasis through immune escape. Nat Med.

[23] Bi X, Hameed M, Mirani N, Pimenta EM, Anari J, Barnes BJ (2011). Loss of interferon regulatory factor 5 (IRF5) expression in human ductal carcinoma correlates with disease stage and contributes to metastasis. Breast Cancer Res.

[24] Sistigu A, Yamazaki T, Vacchelli E, Chaba K, Enot DP, Adam J (2014). Cancer cell-autonomous contribution of type I interferon signaling to the efficacy of chemotherapy. Nat Med.

[25] Schiavoni G, Sistigu A, Valentini M, Mattei F, Sestili P, Spadaro F (2011). Cyclophosphamide synergizes with type I interferons through systemic dendritic cell reactivation and induction of immunogenic tumor apoptosis. Cancer Res.

[26] Yamazaki T, Kirchmair A, Sato A, Buque A, Rybstein M, Petroni G (2020). Mitochondrial DNA drives abscopal responses to radiation that are inhibited by autophagy. Nat Immunol.

[27] Rodriguez-Ruiz ME, Vitale I, Harrington KJ, Melero I, Galluzzi L (2020). Immunological impact of cell death signaling driven by radiation on the tumor microenvironment. Nat Immunol.

[28] Petroni G, Buque A, Coussens LM, Galluzzi L (2022). Targeting oncogene and non-oncogene addiction to inflame the tumour microenvironment. Nat Rev Drug Disco.

[29] Bald T, Landsberg J, Lopez-Ramos D, Renn M, Glodde N, Jansen P (2014). Immune cell-poor melanomas benefit from PD-1 blockade after targeted type I IFN activation. Cancer Disco.

[30] Kepp O, Marabelle A, Zitvogel L, Kroemer G (2020). Oncolysis without viruses - inducing systemic anticancer immune responses with local therapies. Nat Rev Clin Oncol.

[31] Zhou H, Forveille S, Sauvat A, Yamazaki T, Senovilla L, Ma Y (2016). The oncolytic peptide LTX-315 triggers immunogenic cell death. Cell Death Dis.

[32] Yamazaki T, Wennerberg E, Hensler M, Buque A, Kraynak J, Fucikova J (2021). LTX-315-enabled, radiotherapy-boosted immunotherapeutic control of breast cancer by NK cells. Oncoimmunology.

[33] Talpaz M, Mercer J, Hehlmann R (2015). The interferon-alpha revival in CML. Ann Hematol.

[34] Jiang H, Liu XH, Kong J, Wang J, Jia JS, Lu SY (2021). Interferon-alpha as maintenance therapy can significantly reduce relapse in patients with favorable-risk acute myeloid leukemia. Leuk Lymphoma.

[35] Magenau JM, Peltier D, Riwes M, Pawarode A, Parkin B, Braun T (2021). Type 1 interferon to prevent leukemia relapse after allogeneic transplantation. Blood Adv.

[36] Swatler J, Turos-Korgul L, Kozlowska E, Piwocka K. Immunosuppressive Cell Subsets and Factors in Myeloid Leukemias. Cancers (Basel) 2021;13. 10.3390/cancers13061203. 10.3390/cancers13061203 PMC7998753 33801964

[37] Hope KJ, Jin L, Dick JE (2004). Acute myeloid leukemia originates from a hierarchy of leukemic stem cell classes that differ in self-renewal capacity. Nat Immunol.

[38] Anguille S, Lion E, Willemen Y, Van Tendeloo VF, Berneman ZN, Smits EL (2011). Interferon-alpha in acute myeloid leukemia: an old drug revisited. Leukemia.

[39] Bernard JJ, Cowing-Zitron C, Nakatsuji T, Muehleisen B, Muto J, Borkowski AW (2012). Ultraviolet radiation damages self noncoding RNA and is detected by TLR3. Nat Med.

[40] Baessler T, Charton JE, Schmiedel BJ, Grunebach F, Krusch M, Wacker A (2010). CD137 ligand mediates opposite effects in human and mouse NK cells and impairs NK-cell reactivity against human acute myeloid leukemia cells. Blood.

[41] Baessler T, Krusch M, Schmiedel BJ, Kloss M, Baltz KM, Wacker A (2009). Glucocorticoid-induced tumor necrosis factor receptor-related protein ligand subverts immunosurveillance of acute myeloid leukemia in humans. Cancer Res.

[42] Coles SJ, Wang EC, Man S, Hills RK, Burnett AK, Tonks A (2011). CD200 expression suppresses natural killer cell function and directly inhibits patient anti-tumor response in acute myeloid leukemia. Leukemia.

[43] Benjamin R, Khwaja A, Singh N, McIntosh J, Meager A, Wadhwa M (2007). Continuous delivery of human type I interferons (alpha/beta) has significant activity against acute myeloid leukemia cells in vitro and in a xenograft model. Blood.

[44] Zhu C, Zou C, Guan G, Guo Q, Yan Z, Liu T (2019). Development and validation of an interferon signature predicting prognosis and treatment response for glioblastoma. Oncoimmunology.

[45] Snijders AM, Langley S, Mao JH, Bhatnagar S, Bjornstad KA, Rosen CJ (2014). An interferon signature identified by RNA-sequencing of mammary tissues varies across the estrous cycle and is predictive of metastasis-free survival. Oncotarget.

[46] Galluzzi L, Kroemer G (2022). Immuno-epigenetic escape of cancer stem cells. Nat Immunol.

[47] Musella M, Guarracino A, Manduca N, Galassi C, Ruggiero E, Potenza A (2022). Type I IFNs promote cancer cell stemness by triggering the epigenetic regulator KDM1B. Nat Immunol.

[48] Smits EL, Anguille S, Berneman ZN (2013). Interferon alpha may be back on track to treat acute myeloid leukemia. Oncoimmunology.

[49] Dagorne A, Douet-Guilbert N, Quintin-Roue I, Guillerm G, Couturier MA, Berthou C (2013). Pegylated interferon alpha2a induces complete remission of acute myeloid leukemia in a postessential thrombocythemia myelofibrosis permitting allogenic stem cell transplantation. Ann Hematol.

