# Supplementary material for: Type I interferon signaling in malignant blasts contributes to treatment efficacy in AML patients
Source: Cell Death Dis. 2023 Mar 24;14(3):209. doi: 10.1038/s41419-023-05728-w (PMC10039058; doi:10.1038/s41419-023-05728-w)
Supplement: Supplementary file 1 — Supplemental data [file 41419_2023_5728_MOESM1_ESM.pdf]

Supplemental Material and Methods

RNA isolation and reverse transcription. Total RNA was isolated from the peripheral blood mononuclear cells (PBMCs) of healthy donors (HDs) or AML patients (upon depletion of red blood cells using the ACK lysing buffer, from Thermo Fisher Scientific) as well as from isolated $CD33^+$ malignant blasts, using the RNeasy Mini Kit (Qiagen) as per the manufacturer's recommendations. RNA concentration and purity were determined using a NanoDrop 2000c (Thermo Scientific), while RNA integrity was assessed using an Agilent 2000 Bioanalyzer (Agilent). Purified RNA samples were stored at -80°C, and only samples with an integrity index ≥ 7 were subsequently used for reverse transcription and RNAseq. The cDNA for the quantification by RT-qPCR was synthesized from total RNA using the iScript cDNA Synthesis Kit (Bio-Rad).

Bulk RNA sequencing and analysis. Sequencing libraries were created using NEBNext Poly(A) mRNA Magnetic isolation Module and NEBNext Ultra II Directional RNA Library Prep kits (both New England BioLabs) and were sequenced by NovaSeq 6000 SP (Illumina). Raw FASTQ sequencing files were aligned to human reference genome (build h19) with bowtie2 (version 2.3.2) and tophat2 (version 2.1). Expression levels as raw "counts" were calculated from aligned reads with mapping quality ≥10 using htseq-count (version 0.6.0). Differential gene expression analyses were performed using DESeq2 (version 1.24.0) in R. Heatmaps were assembled using the ComplexHeatmap package in R.

The Cancer Genome Atlas program. The normalized gene expression data for 152 AML patients (after M3 and M7 AML subtypes exclusion) were retrieved from the public database (TCGA) and were used in correlation analyses.

Cell lines. KASUMI-1 and MOLM13 cells were grown in RPMI 1640 Medium containing 4 mM *L*-glutamine and 4500 mg/L glucose (Gibco), and supplemented with 20% fetal bovine serum (FBS,

from PAA) and 100 U/mL penicillin plus 100 µg/mL streptomycin (Gibco). MV4-11 and K562 cells were cultured in IMDM containing 4 mM *L*-glutamine and 4500 mg/L glucose (Gibco) and supplemented with 10% FBS and 100 U/mL penicillin plus 100 µg/mL streptomycin. PBMCs and $CD33^+$ blasts were cultured in RPMI 1640 Medium containing 4 mM *L*-glutamine and 4500 mg/L glucose and supplemented with 10% plasma derived human pooled serum (PHS, from Thermo Fisher) and 100 U/mL penicillin plus 100 µg/mL streptomycin. All cell lines were regularly tested for *Mycoplasma* contamination using the MycoAlert® Mycoplasma Detection Kit (Lonza) and Spark reader (Tecan) to detect luminescence. Cell lines were used for experiments 2 to 5 passages from thawing and were maintained at 30-70% confluency.

Multiplex assay. Concentration of IFN-α2 protein in the sera of AML patients was assessed by MAGPIX system (Luminex) using Magnetic Bead Panel HCYTOMAG-60K, 6-plex (Merck) according to manufacturer´s protocol.

ELISA. Concentration of IFN-β in cell culture supernatant was determined using High sensitivity human IFN Beta TCM ELISA (PBL) or by Human IFN-beta DuoSet ELISA (R&D) according to manufacturer's protocol. Sample absorbance was measured by Sunrise Microplate reader (Tecan).

[^{3}H]-thymidine *incorporation assay*. Inhibition of cell proliferation was determined using the [^{3}H]-thymidine incorporation assay. The cells (2 x 10^4/well) were seeded into a 96-well flat-bottom tissue culture plate (Nunc) and were cultivated in growth medium in final volume of 250uL. The plates were incubated in a 5% CO2 atmosphere at 37°C. Triplicates to pentaplets were used for each test condition. At the beginning of the assay cells were incubated with rIFN-α and rIFN-β (500pg/mL) for 7 days and each well was pulsed with 1 µCi (37 kBq) of [^{3}H]-thymidine for the last 6–8 h of total incubation time. The cells were then harvested on glass fiber filters (Perkin Elmer) using a cell

harvester (Tomtec) and the radioactive emission was measured in a scintillation counter (1450 Microbeta TriLux, Wallac).

Colony-forming assays. For colony-forming assays, 5 x 10^3 $CD34^+$ enriched cells were cultured in 6-well plates in a mixture of 300 µL IMDM plus 2700 µL of the semi-solid methylcellulose-based media MethoCult™ H4534 Classic Without EPO (StemCell Technologies). Colonies were quantified according to the manufacturer´s guidelines. LSCs were cultured and maintained using a specific medium that preserves the LSC phenotype: StemSpan™ Leukemic Cell Culture Kit (StemCell Technologies).

Preparation of knockout cell line. KASUMI-$1^{IFNAR2-/-}$ cell lines was prepared by Crispr-Cas9 guided gene targeting technology. Two gRNA were designed with aim to delete exon three, specifically g353 targeting exon two and g1716 targerting 3´end of exon three (**Supplemental Table 4)**. KASUMI-1 cells were electroporated with mixture of gRNAs incorporated in pSpCas9(BB)-2A-GFP (PX458) expression vector for dual expression of Cas9 and gRNA. Two days after electroporation, single GFP^+ cells were single cell sorted into 96wp coated with bone marrow cells of NSG mice and expanded. Thereafter, genomic DNA from single clones was extracted and PCR amplification of the genomic gRNA target sites was performed. Of all 14 clones analyzed, none exhibited deletion of exon three that would otherwise result in the presence of 1.4kb PCR fragment, the only PCR fragment detected was wt-like 2.2kb (**Supplemental Fig. 2A**). Next, we chose several clones and performed sequencing of gRNA target regions. In contrast to parental KASUMI-1 cells, some clones provided consistent sequence reads only to the expected break site (DSB) indicating presence of multiple sequences downstream of DSB caused by presence of indels (**Supplemental Fig. 2B**). Several clones were analyzed for expression of IFNAR2 by FACS using APC-labelled anti IFNAR2 antibody (**Supplemental Table 3**). Several clones do not express membrane bounds IFNAR2 compared to KASUMI-1 cells (**Supplemental Fig. 2 C**). For further studies, clone D was

selected based on similarity in response to daunorubicin (**Supplemental Fig. 2D**), proliferation rates (data not shown) to parental cell line and no expression of IFNAR2 protein as shown by flow cytometry. Confirmation that both IFNAR2 alleles were successfully targeted, we performed TA cloning (pGEM®-T Easy, Promega) followed by sequencing of gRNA target regions of clone D. In total we analysed 13 clones and identified two different DNA repair mechanisms for both g352 and g1716: for g352 insertion of GG and indel (deletion of six nucleotides combined with insertion of CTC), for g1716 insertions, CGTACAAC and GCCA in DSB site were found. Moreover, sequencing revealed that all four mutations were combined with each other resulting in 4 different clones. We therefore presumed that established culture originates from two clones, however none of them has DNA sequence reads at gRNA target region corresponding to parental WT cells. All insertions have changed reading frame or have removed splice donor site of exon 3 (CGTACAAC insertion in g1716) resulting in creation of premature stop codon downstream, indel in g352 keeps the reading frame, however, it was combined with stop-codon creating mutations in g1716. We can conclude that, that in clone D, IFNAR2 gene was homozygously targeted, resulting in complete gene knock-out.

Preparation of cell lysates and immunoblotting analysis

Cells were harvested and washed with ice-cold PBS and subsequently lysed in sample buffer (300 mM Tris pH 6.8, 5% SDS, 50% Glycerol, 360 mM β-Mercaptoethanol, 0.05% Bromophenol blue). Proteins were separated by 11% sodium dodecyl-sulfate polyacrylamide gel electrophoresis (SDS-PAGE) and then transferred to 0.45μm nitrocellulose membranes (Biorad). Efficiency of a protein transfer was checked by Ponceau-S staining (Abcam). The membranes were blocked in 5% nonfat dry milk in TBST buffer (50 mM Tris, 150 mM NaCl, 0.05% Tween 20) for 1h at room temperature and incubated with primary antibody overnight at 4°C. Membranes were washed in TBST buffer and incubated for 1h at room temperature with relevant horseradish peroxidase-conjugated secondary antibodies. SuperSignal West Dura Extended duration detection system (ThermoFisher) was used for a protein visualization, performer by imaging system ChemiDoc (Biorad).

Mice and in vivo experiments. Rag2$^{-/-}$ mice on BALB/c background (*H-2^{d}*) used for *in vivo* experiments were 8-20 weeks old and with body weight at least 19 g. Mice were obtained from the Institute of Microbiology of the Czech Academy of Sciences (Prague, Czech Republic) breeding colony. Food and water were given *ad libitum*. All animal work strictly followed the protocol approved by the Institutional Animal Care and Use Committee of the Czech Academy of Sciences, with all experiments conducted in compliance with local and European guidelines. Rag2$^{-/-}$ mice were i.v. inoculated with 2.5 x 10^{6} KASUMI-1^{WT} or KASUMI-1$^{IFNAR2-/-}$ cells in 300 µL PBS on day 0. Human rIFN-β was i.p. injected on days 13, 14, 15 and 16 twice daily (early morning and late afternoon) in 250 µL PBS using 1.5 µg/dose, i.e. 3 ug per mouse each day. Daunorubicin was administered via tail vein injection on days 15, 17 and 19 in 300 µL PBS using 4 mg/kg per one dose. Body weight of experimental mice was recorded 2-3 times a week throughout the whole experiment.

Supplemental Figures

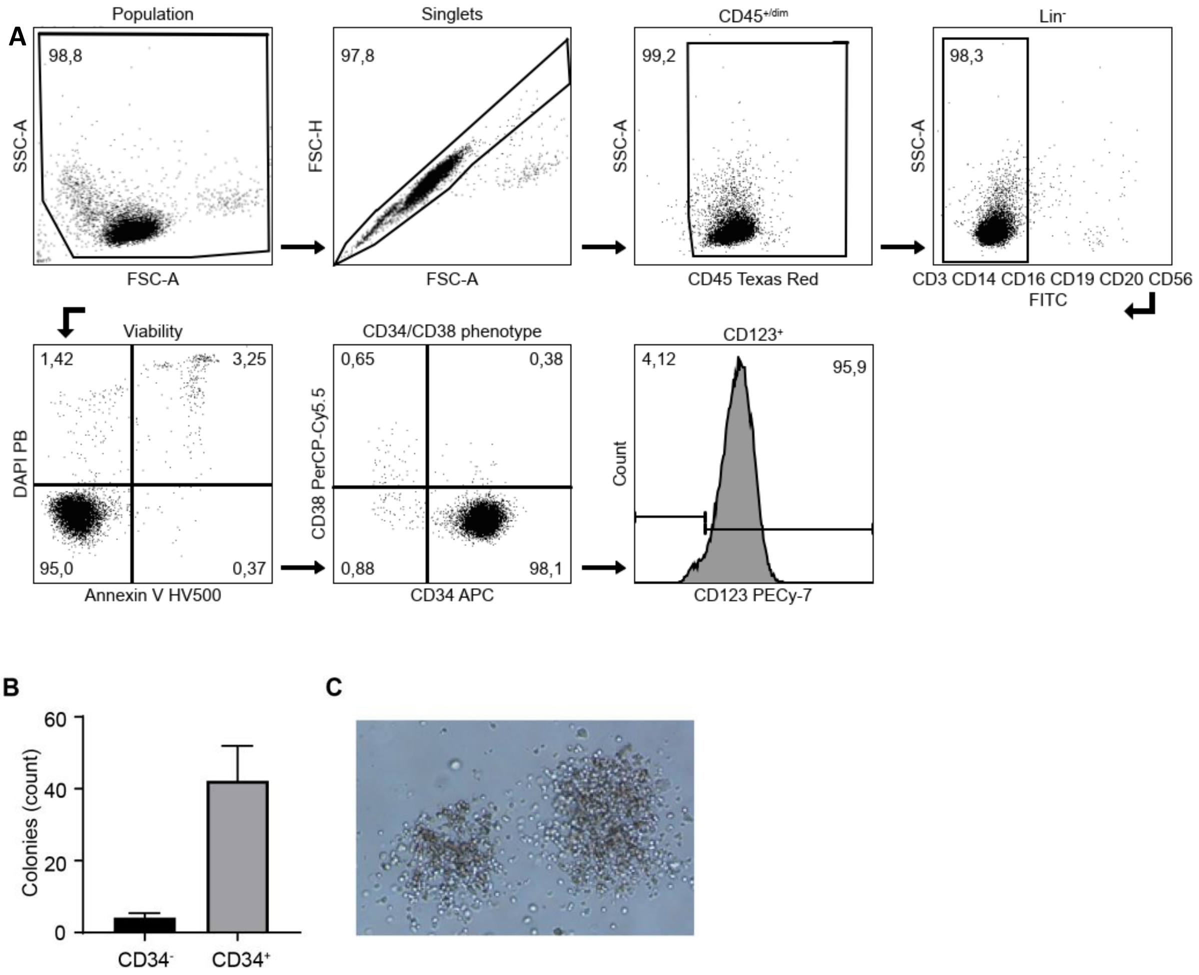

Supplemental Figure 1. **Isolation of leukemic stem cells (LSCs).** (**A**) Gating strategy for CD45^{dim}, Lin^{-} (CD3^{-}CD14^{-}CD16^{-}CD19^{-}CD20^{-}CD56^{-}), CD34^{+}, CD38$^{+/-}$ and CD123$^{+/dim}$ LSCs phenotype on isolated CD34^{+} cells of AML patients. (**B**) Number of colonies formed by CD34^{+} versus CD34^{-} fraction obtained by CD34^{+} magnetic purification from 10 AML patients (study cohort 1) assessed by bright field microscopy (**C**) The representative image of LSC colonies.

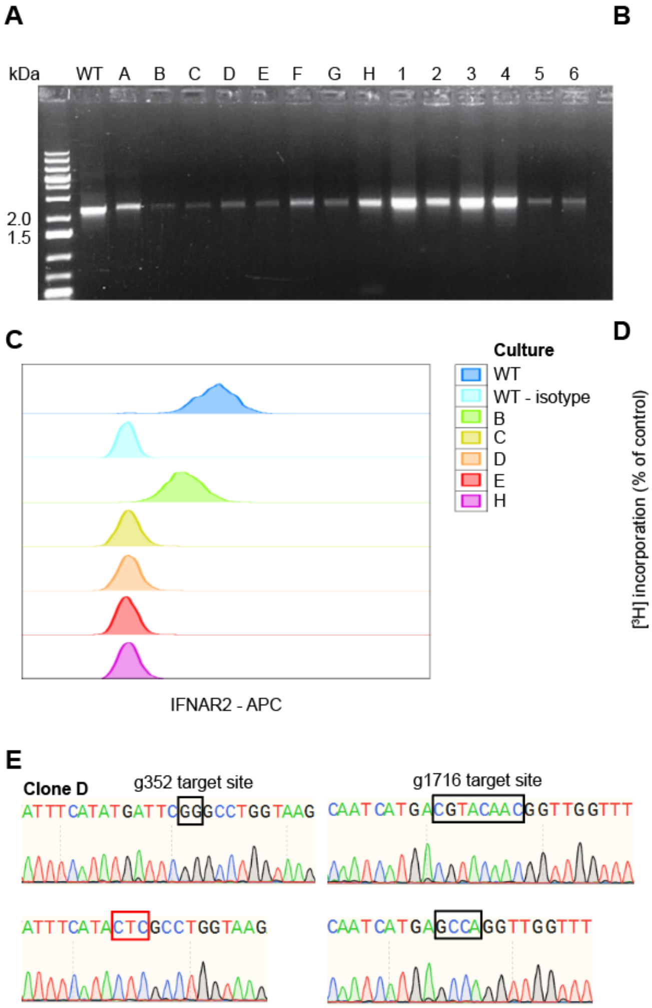

Supplemental Figure 2. **Generation of KASUMI-1$^{IFNAR2-/-}$ cell line.** (**A**) PCR products of a target region of *IFNAR2* gene using mixture of g352 and g1716. (**B**) Sequencing of genomic gRNA target sites of several clones and WT KASUMI-1 cells. (**C**) Expression of *IFNAR2* determined by flow cytometry. (**D**) Response of selected clones and WT culture to daunorubicin as analyzed by [^{3}H]-thymidine incorporation. (**E**) Sequencing of gRNA target site of clone D.

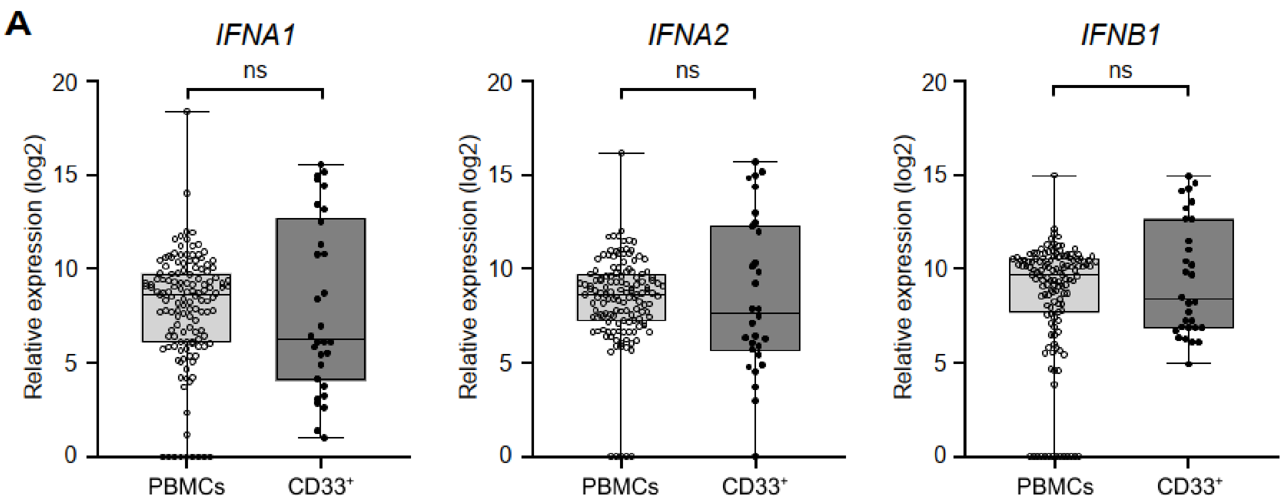

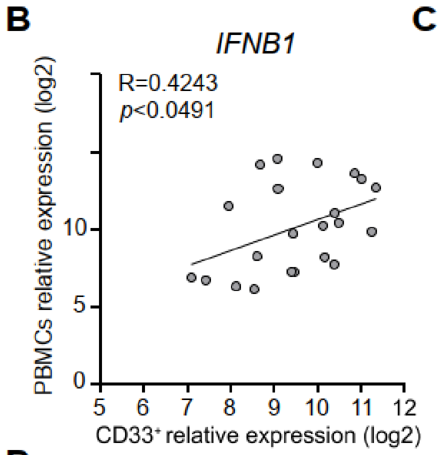

C

GENE		*cGAS*	*DDX58*	*IFIH1*	MAVS	*EIF2AK*	*STING1*	*TLR3*	*TLR7*	*TLR9*	*ZBP1*
IFNA1	R	0,06	-0,13	0,09	-0,01	0,10	0,04	0,77	0,13	0,56	-0,41
	p	0,75	0,51	0,62	0,95	0,61	0,82	<0,0001	0,49	<0,01	0,03
IFNA2	R	0,28	0,01	0,08	-0,02	-0,13	0,15	0,57	-0,02	0,38	-0,46
	p	0,15	0,95	0,66	0,54	0,98	0,69	<0,01	0,91	0,07	0,01
IFNB1	R	0,17	-0,03	0,08	0,19	0,18	0,12	0,79	-0,02	0,55	-0,36
	p	0,37	0,86	0,66	0,32	0,35	0,51	<0,0001	0,92	<0,01	0,06
IFN-i	R	0,06	-0,13	0,09	-0,01	0,10	0,04	0,67	0,13	0,56	-0,41
	p	0,75	0,51	0,62	0,95	0,61	0,82	<0,0001	0,49	<0,01	0,03

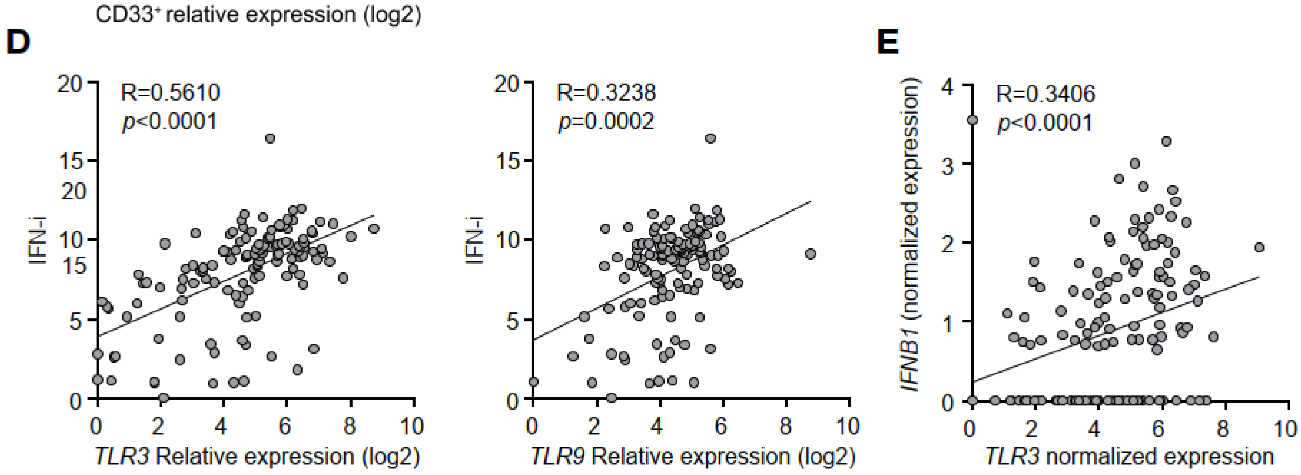

Supplemental Figure 3. **TLR3 drives type I IFN secretion from AML blasts. (A)** Relative expression level of *IFNA1*, *IFNA2*, and *IFNB1* in peripheral blood of AML patients (n=132) and in isolated $CD33^+$ malignant blasts (n=30) (study cohort 1), as determined by RT-qPCR. Statistical significance was calculated by the Mann-Whitney test. ns: not significant. plots: lower quartile, median, upper quartile; whiskers, minimum, maximum. (**B**) Correlation between *IFNB1* expression

levels in peripheral blood and isolated CD33$^+$ malignant blasts in 22 paired AML patient´s samples; R, Pearson´s correlation coefficient. (**C**) Correlation between type I IFN genes (*IFNA1, IFNA2, IFNB1*), IFN index (IFN-i) and *CGAS, DDX58, IFIH1, MAVS, EIF2AK, STING1, TLR3, TLR7, TLR9* and *ZBP1* in isolated CD33$^+$ malignant blasts (n=30) from AML patients. Pearson´s correlation coefficient or Spearman´s correlation coefficient, R and *p* (also see Figure 1E**).** (**D**) Correlation between IFN-i and *TLR3* and *TLR9* expression levels in peripheral blood of 132 AML patients (study cohort 1). R, Spearman´s correlation coefficient. (**E**) Correlation between *IFNB1* and *TLR3* expression levels of 152 AML patients retrieved from TCGA public database (study cohort 2). R, Spearman´s correlation coefficient.

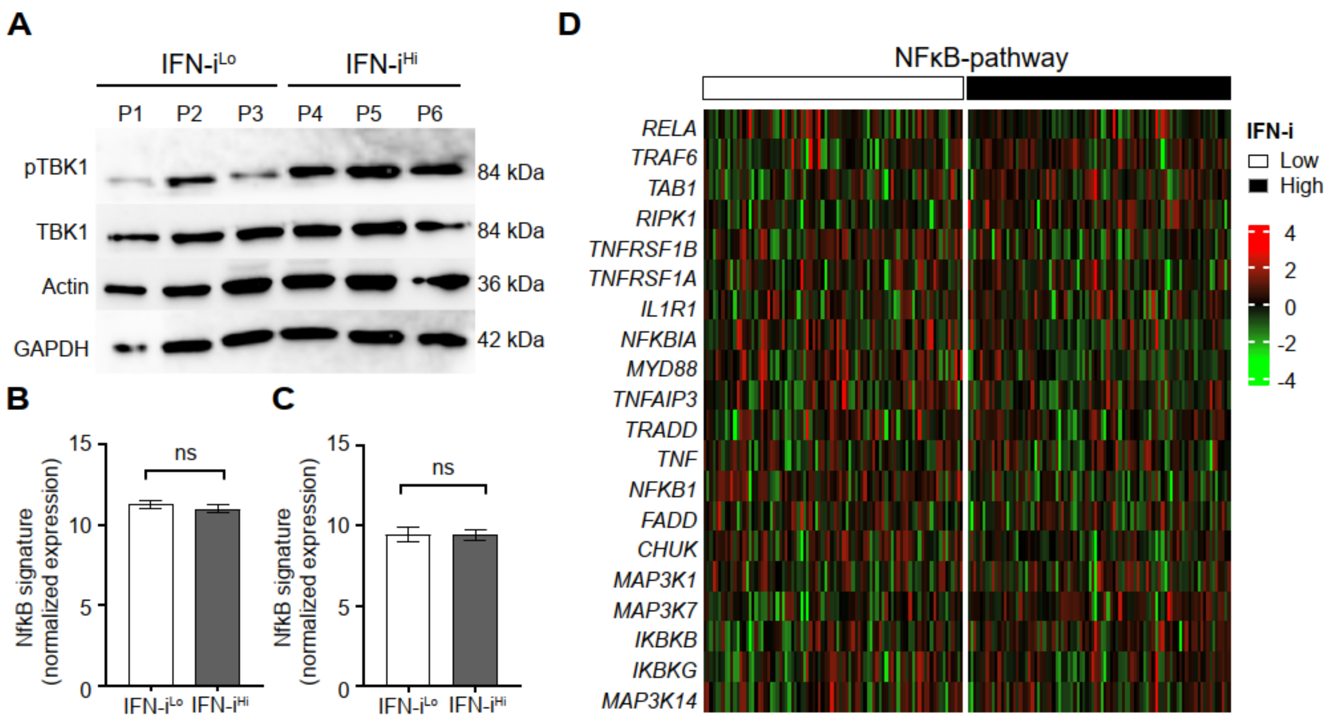

***Supplemental Figure 4*. Activation of TBK1 signaling pathway downstream of TLR3 in IFN-i^{Hi} AML patients.** Phosphorylation of TBK1 in CD33^{+} malignant blasts of 3 IFN-i^{Lo} and 3 IFN-i^{Hi} AML patients from study cohort 1 determined by immunoblot detection of phosphorylated TBK1, TBK1 irrespective of its phosphorylation state, using actin and GAPDH as loading control **(A)**. **(B-D)** Relative expression levels and heatmap of gene signature associated with NFκB signaling pathway in 12 type I IFNi^{Lo} (n=12) and IFN-i^{Hi} (n=12) patients from study cohort 1 **(B)** and in type I IFN-i^{Lo} (n=76) and IFN-i^{Hi} (n=76) AML patients from TCGA public database determined by RNAseq **(C, D)**. Data are presented as median. Significant *p* values are reported; ns, not significant (Mann-Whitney test).

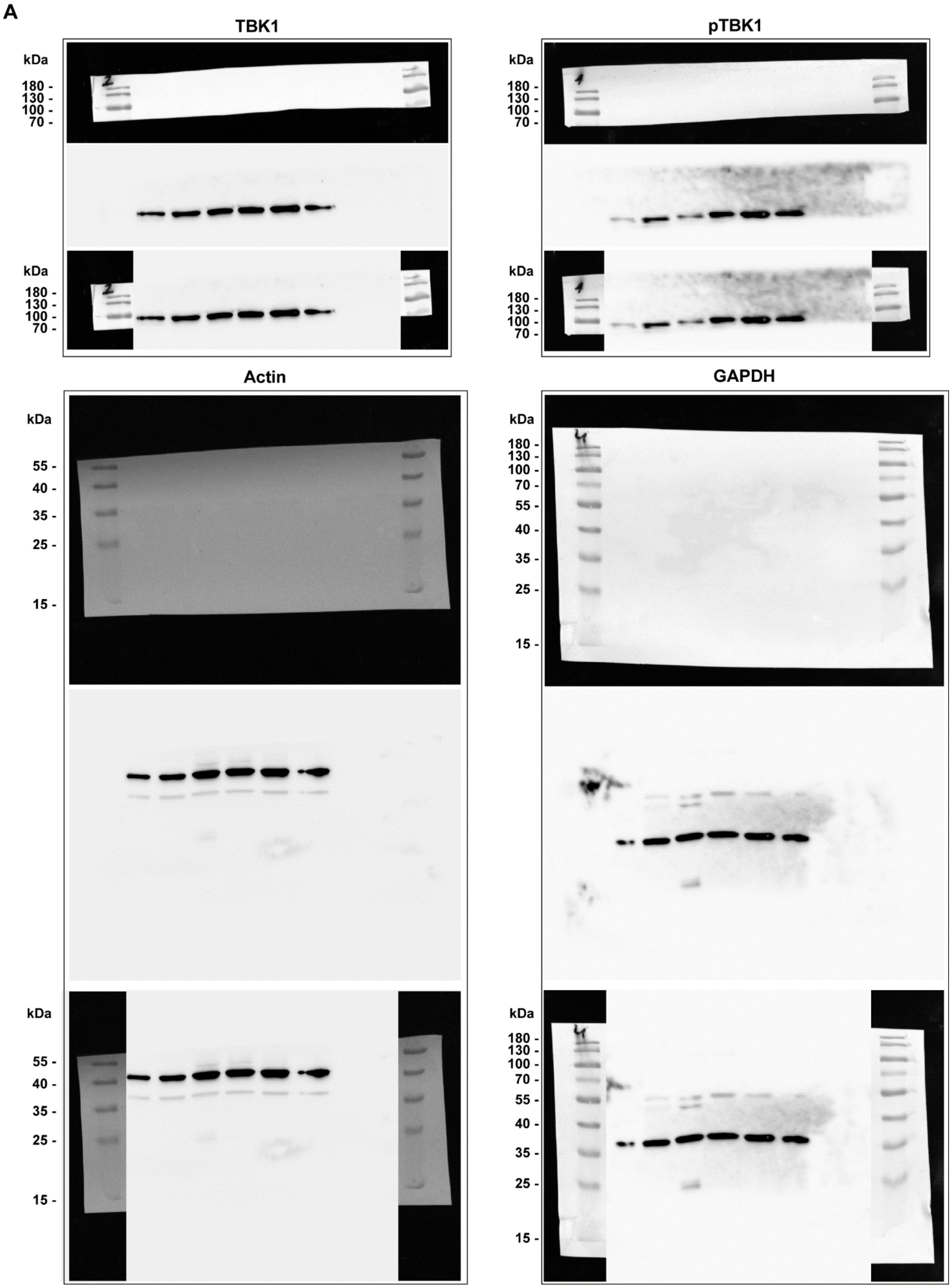

Supplemental Figure 5. **(A) Uncropped full-length images of western blot membranes** (see Supplementary Figure 4A)

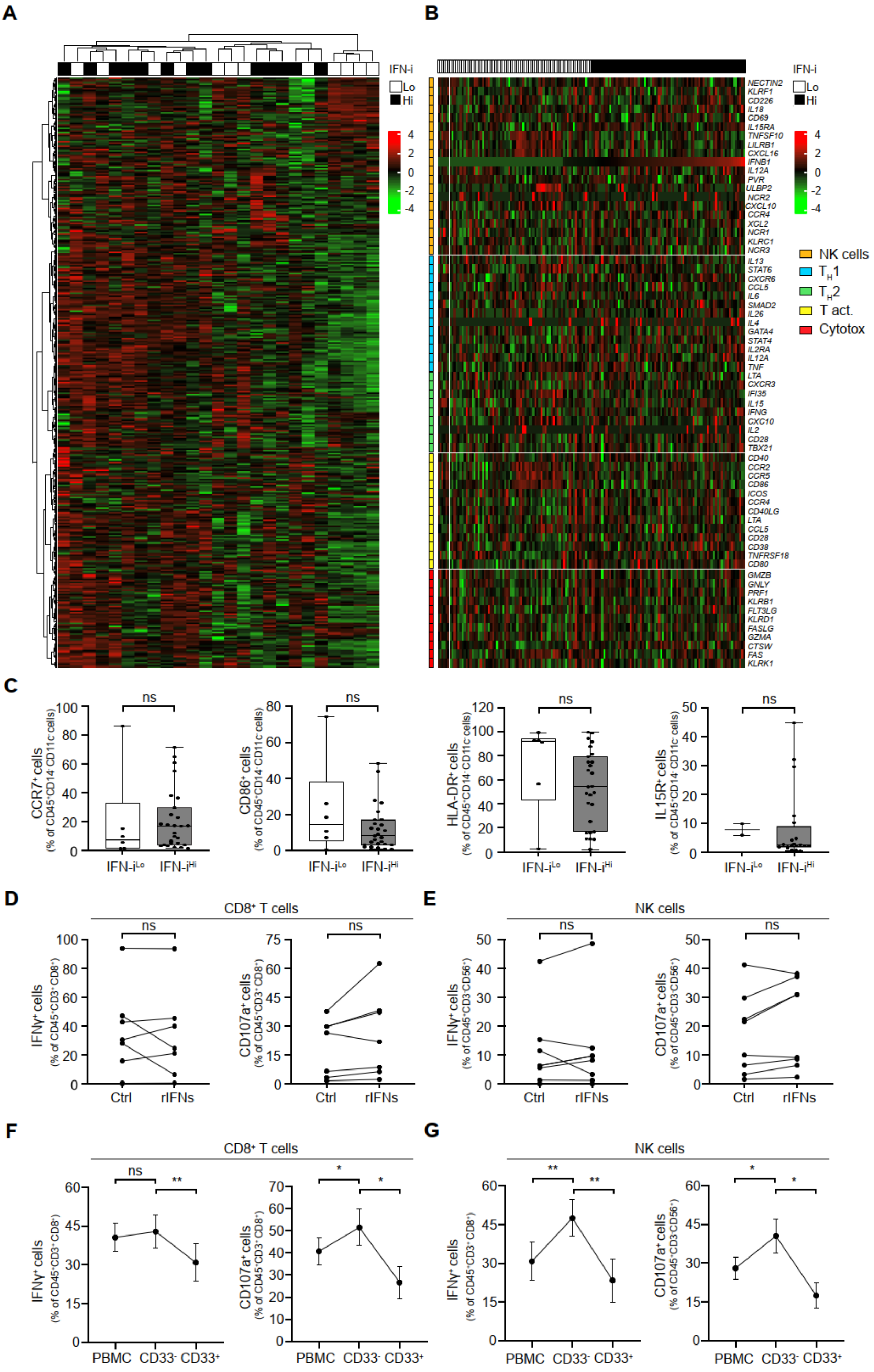

A
IFN-i
Lo
Hi
B
IFN-i
Lo
Hi
NK cells
TH1
TH2
T act.
Cytotox
NECTIN2
KLRF1
CD226
IL18
CD69
IL15RA
TNFSF10
LILRB1
CXCL16
IFNB1
IL12A
PVR
ULBP2
NCR2
CXCL10
CCR4
XCL2
NCR1
KLRC1
NCR3
IL13
STAT6
CXCR6
CCL5
IL6
SMAD2
IL26
IL4
GATA4
STAT4
IL2RA
IL12A
TNF
LTA
CXCR3
IFI35
IL15
IFNG
CXC10
IL2
CD28
TBX21
CD40
CCR2
CCR5
CD86
ICOS
CCR4
CD40LG
LTA
CCL5
CD28
CD38
TNFRSF18
CD80
GMZB
GNLY
PRF1
KLRB1
FLT3LG
KLRD1
FASLG
GZMA
CTSW
FAS
KLRK1
C
CCR7+ cells (% of CD45+CD14-CD11c- cells)
CD86+ cells (% of CD45+CD14-CD11c- cells)
HLA-DR+ cells (% of CD45+CD14-CD11c- cells)
IL15R+ cells (% of CD45+CD14-CD11c- cells)
ns
IFN-iLo
IFN-iHi
D
CD8+ T cells
IFNγ+ cells (% of CD45+CD3+CD8+)
CD107a+ cells (% of CD45+CD3+CD8+)
Ctrl
rIFNs
E
NK cells
IFNγ+ cells (% of CD45+CD3-CD56+)
CD107a+ cells (% of CD45+CD3-CD56+)
F
CD8+ T cells
IFNγ+ cells (% of CD45+CD3+CD8+)
CD107a+ cells (% of CD45+CD3+CD8+)
PBMC
CD33-
CD33+
G
NK cells
IFNγ+ cells (% of CD45+CD3+CD8+)
CD107a+ cells (% of CD45+CD3-CD56+)

Supplemental Figure 6. **Type I IFN-driven immunostimulation is suppressed by malignant blasts.** (**A**) Hierarchical clustering of 433 transcripts that were significantly changed in IFN-i^{Lo} vs IFN-i^{Hi} peripheral blood samples from study cohort 1, as determined by RNA sequencing. (**B**) Expression levels of selected genes associated with NK cells, T_H1 and T_H2 response, T cell activation and cytotoxicity in 76 *IFNB1*^{Lo} and 76 *IFNB1*^{Hi} AML patients from study cohort 2. (**C**) Percentage of circulating CCR7$^+$, CD86$^+$, HLA-DR$^+$ CD14$^+$CD11c$^+$ cells in 6 IFN-i^{Lo} versus 27 IFN-i^{Hi} AML patients from study cohort 1 prior induction chemotherapy. Statistical significance was calculated by the Mann-Whitney test. Box plots: lower quartile, median, upper quartile; whiskers, minimum value, maximum value; ns: p value>0,05. A paired comparison of the percentage of IFN-γ^+ and CD107a$^+$ CD8$^+$ T cells (**D**) and NK cells (**E**) upon PMA/Ionomycin stimulation in peripheral blood of AML patients (study cohort 1; n=7) with/without pre-incubation with rIFN, as determined by flow cytometry. A paired comparison of IFN-γ^+ and degranulating CD107a$^+$ CD8$^+$ T cells **(F)** and NK cells **(G)** after PMA/Ionomycin stimulation of 10 AML patient PBMCs before, after CD33$^+$ malignant blasts separation (CD33$^-$) and after re-addition of viable autologous CD33$^+$ malignant blasts using PMA/Ionomycin for stimulation. Statistical significance was calculated by the Wilcoxon test. p values are indicated.

A

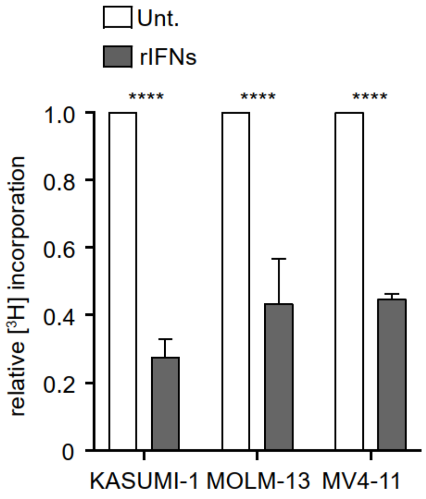

Supplemental Figure 7. **Recombinant type I IFN mediates direct cytostatic activity on AML cell lines.** (**A**) Proliferation capacity of KASUMI-1, MOLM-13 and MV4-11 cells after 24h treatment with recombinant IFNs (rIFNs) determined by incorporation of [^{3}H]-thymidine. Statistical significance was calculated by the paired T-test; Data are reported as a fold change relative to control cells; bars represent the means of three independent experiment; whiskers: standard deviation. ***p<0.001 (paired t-test).

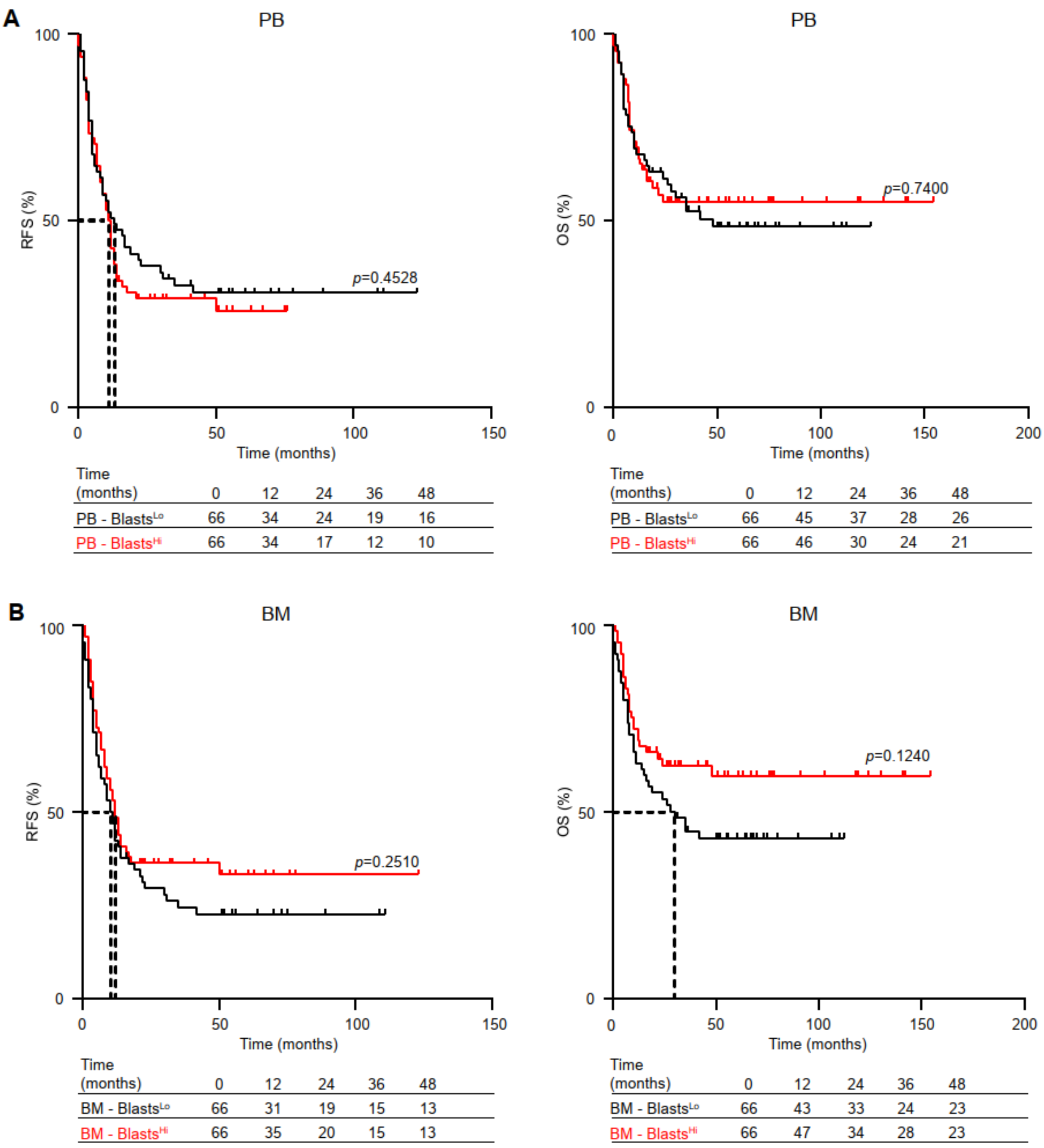

Supplemental Figure 8. **The clinical relevance of malignant blasts frequency in peripheral blood.** (**A, B**) Relapse-free (RFS) and overall survival (OS) of 132 AML patients from study cohort 1 stratified into two groups based on median value of frequency of malignant blasts in peripheral blood (**A**) or in bone marrow (**B**). Survival curves were estimated by the Kaplan-Meier method, and differences between groups were evaluated using log-rank test. Number of patients at risk and *p* values are reported.

Supplemental tables

Supplemental Table 1. The main clinical and biological characteristics of 16 AML patients from Study Cohort 3.

Variable	Study cohort n=16
Age at diagnosis	
< 50 years	6 (37%)
≥ 50 years	10 (63%)
Median (years)	51
Range (years)	39-68
Sex	
Male	3 (19%)
Female	13 (81%)
Peripheral-blood white cell count	
< 30.000/mm^3	10 (63%)
≥ 30.000/mm^3	6 (37%)
Median (10^9 cells/l)	27,4
Range (10^9 cells/l)	2,16-375,8
Blasts peripheral blood	
Median (%)	42
Range (%)	0,9-96
Blasts bone marrow	
Median (%)	54
Range (%)	23-95
De novo AML	16 (100%)
Secondary AML	
MDS/MPN, *n*	0 (0%)
Therapy related, *n*	0 (0%)
Not specified, *n*	0 (0%)
FAB classification	
M0	0 (0%)
M1	3 (19%)
M2	6 (38%)
M4	7 (43%)
M5	0 (0%)
M6	0 (0%)
Cytogenetic profile	
Favorable	2 (12,5%)
Intermediate	12 (75%)
Adverse	2 (12,5%)
Missing data	0 (0%)

Molecular characteristics	
DNMT3A	3 (19%)
FLT3-ITD	3 (19%)
FLT3-TKD	3 (19%)
CBFβ::MYH11	1 (6%)
NPM1	5 (31%)
IDH2	4 (25%)
Induction chemotherapy	
Daunorubicin + Ara-C (3+7)	3 (19%)
Idarubicin + Ara-C (3+7)	13 (81%)
BIDFA	0 (0%)
FLA-IDA	0 (0%)
HAM	0 (0%)
**Complete remission**	15 (94%)
Consolidation	
Chemotherapy only	5 (31%)
HSCT	11 (69%)

Abbreviations: AML, acute myeloid leukemia; Ara-C, cytarabine (well known as cytosine arabinoside); BIDFA, combination of twice daily fludarabine and cytarabine; CBFβ::MYH11, Core binding factor β - Myosin Heavy Chain 11 fusion protein ; DNMT3A, DNA (cytosine-5)-methyltransferase 3A; FAB, French-American-British; FLA-IDA, fludarabine-idarubicin; FLT3-ITD, Fms Related Receptor Tyrosine Kinase - internal tandem duplication; FLT3-TKD, Fms Related Receptor Tyrosine Kinase – tyrosine kinase domain mutation; HAM, high-dose cytosine arabinoside and mitoxantrone; HSCT, hematopoietic stem cell transplantation; IDH2, Isocitrate dehydrogenase 2 ($NADP^+$); MDS, myelodysplastic syndrome; MPN, myeloproliferative neoplasm; NPM1, nucelophosmin 1.

Supplemental Table 2. Primers and probes with sequences used for RT-qPCR.

Gene Name	Sequence 5´- 3´	Company
**CGAS_fw**	GACTGAAGTGCGACTCCG	Generi Biotech
**CGAS_probe**	6FAM-TGCTGAACACCGGGAGCTACTATGA--BBQ	Generi Biotech
**CGAS_rev**	TGACATCAAATTCATTAGGTGCAG	Generi Biotech
**DDX58 probe**	6FAM-AGAACACAGGAATGACCCTCCCG--BBQ	Generi Biotech
**DDX58_fw**	CCACTGGCTTTGAATGCATC	Generi Biotech
**DDX58_rev**	TGGCATATTGACTGGACGTG	Generi Biotech
**EIF2AK2_fw**	GCGAACAAGGAGTAAGGGAAC	Generi Biotech
**EIF2AK2_probe**	6FAM-TGCGATACATGAGCCCAGAACAGATTT--BBQ	Generi Biotech
**EIF2AK2_rev**	AATTAGCCCCAAAGCGTAGAG	Generi Biotech
**IFIH1_probe**	6FAM-TGAGAGCACCTACGTCCTGGTTG--BBQ	Generi Biotech
**IFIH1_fw**	CGTCACCAATGAAATAGCCATG	Generi Biotech
**IFIH1_rev**	TTCGATAACTCCTGAACCACTG	Generi Biotech
**IFNA1_fw**	CATCTggTCCAACATgAAAACAA	TIB MOLBIOL
**IFNA1_probe**	6FAM-CATgAAAgCgTgACCTggTgTATgAgTC--BBQ	TIB MOLBIOL
**IFNA1_rev**	gggTgAgAgTCTTTgAAATgACAg	TIB MOLBIOL
**IFNA2_fw**	TGACTTGCAGCTGAGCAC	Generi Biotech
**IFNA2_probe**	6FAM-ACCAGTAAAGCAAAGGTCAAGGCCA--BBQ	Generi Biotech
**IFNA2_rev**	CCCATTTCAACCAGTCTAGCAG	Generi Biotech
**IFNB1_fw**	gATgCTCCAgAACATCTTTgC	TIB MOLBIOL
**IFNB1_probe**	6FAM-CCAgCCAgTgCTAgATgAATCTTgTCTgAABBq--BBQ	TIB MOLBIOL
**IFNB1_rev**	TAgCCAggAggTTCTCAACAATAgTC	TIB MOLBIOL
**MAVS_fw**	GATTCTGCCTTACCTGCCC	Generi Biotech
**MAVS_probe**	6FAM-TCACAGCAAGAGACCAGGATCGACT--BBQ	Generi Biotech
**MAVS_rev**	GAAGAGATGCCAGAGGGTG	Generi Biotech
**STING1_fw**	AAAAGGGAATTTCAACGTGGC	Generi Biotech
**STING1_probe**	TCCGATGTAATATGACCATGCCAGCC	Generi Biotech
**STING1_rev**	GTAAGTTCGAATCCGGGCC	Generi Biotech
**SURF1_fw**	AgTACATCgTgACCTggTATgg	TIB MOLBIOL
**SURF1_probe**	6FAM-AgAAATTCCTACgTgggACACCTggT--BBQ	TIB MOLBIOL
**SURF1_rev**	gTAgCACATgATCCAgCATAAAg	TIB MOLBIOL
**TLR3_fw**	AACATCCCTGAGCTGTCAAG	Generi Biotech
**TLR3_probe**	CCACTACCTTTGCAACACTCCACCT	Generi Biotech
**TLR3_rev**	AAAAGTCTCACTGGGAACCC	Generi Biotech
**TLR7_fw**	gTTTCCAATgTggACACTgAAgAgA	Generi Biotech
**TLR7_probe**	F-TTAggAAACCATCTAgCCCCAAggA--Q	Generi Biotech
**TLR7_rev**	CTTgTCTgTgCAgTCCACgAT	Generi Biotech
**TLR9_fw**	CTATAACCGGAACTTCTGCCAG	Generi Biotech
**TLR9_probe**	6FAM-AATAGCCGTGAGCCGGAATCCTG--BBQ	Generi Biotech
**TLR9_rev**	TGAGGTGAGTGTGGAGGTG	Generi Biotech
**ZBP1_fw**	AAACTCCGAAGCCATCCAG	Generi Biotech
**ZBP1_probe**	6FAM-TCTGTCTTGTAATGATGTTCCCGTGTCC--BBQ	Generi Biotech
**ZBP1_rev**	CATTGAAGGGAGGTGGCG	Generi Biotech

Supplemental Table 3. Antibodies used for flow cytometry.

Parameter	Source	Producer	Clone	Fluorochrome	Dilution
**Annexin V**	na	EXBIO	na	APC	1:100
**Annexin V**	na	BD Biosciences	na	HV500	1:100
**IFN-γ**	mouse	eBioscience	4S.B3	PE-Cy7	1:100
**CD3**	mouse	eBioscience	OKT3	FITC	4:100
**CD34**	mouse	eBioscience	4H11	APC	6:100
**CD123**	mouse	Biolegend	6H6	PE-Cy7	2:100
**Granzyme B**	mouse	BD Biosciences	GB11	Brilliant Violet 421	5:100
**Granzyme B**	mouse	eBioscience	GB11	PE	5:100
**CD38**	mouse	Biolegend	HIT2	PerCP-Cy5.5	5:100
**CD33**	mouse	Biolegend	WM53	PE	4:100
**CD3**	mouse	EXBIO	MEM-57	AF700	6:100
**CD4**	mouse	eBioscience	RPA-T4	PE-Cy7	5:100
**CD4**	mouse	Biolegend	OKT4	BV421	5:100
**CD8**	mouse	BD Biosciences	RPA-T8	HV500	5:100
**CD14**	mouse	EXBIO	MEM-15	FITC	6:100
**CD11c**	mouse	EXBIO	BU-15	APC	6:100
**CD16**	mouse	EXBIO	LNK16	FITC	6:100
**CD19**	mouse	EXBIO	LT19	FITC	6:100
**CD20**	mouse	EXBIO	LT20	FITC	6:100
**CD45**	mouse	Life Technologies	HI30	PE-Texas Red	6:100
**CD45**	mouse	EXBIO	MEM-28	PerCP	6:100
**CD56**	mouse	Beckman Coulter	N901	ECD	6:100
**CD56**	mouse	BioLegend	MEM-188	PerCP-Cy5.5	6:100
**CD56**	mouse	EXBIO	MEM-188	FITC	6:100
**CCR7**	mouse	Biolegend	G043H7	PerCP-Cy5.5	6:100
**CD86**	mouse	BD Biosciences	2331(FUN-1)	AF700	6:100
**IL15Ralpha**	mouse	Biolegend	JM-7A4	PE	6:100
**HLA-DR**	mouse	BD Biosciences	L243	PE-Cy7	6:100
**IFNAR2**	Rabbit	Sinobiological	#122	APC	6:100
**CD107a**	mouse	BioLegend	H4A3	FITC	8:100

Supplemental Table 4. gRNA targets and primers used in CRISP/Cas9 guided gene therapy.

Target/Primer	Sequence
gRNA352 target	GGTATTTCATATGATTCGCC TGG
**g352 sense (5'-3')**	CACCGGTATTTCATATGATTCGCC
**g352 antisense (5'-3')**	AAACGGCGAATCATATGAAATACC
gRNA1716 target	GCTGTATACAATCATGAGGT TGG
**g1716 sense (5'-3')**	CACCGCTGTATACAATCATGAGGT
**g1716 antisense (5'-3')**	AAACACCTCATGATTGTATACAGC

Supplemental Table 5. Antibodies used for immunoblotting analyses.

Parameter	Source	Producer	Clone	Diluation	Secondary antibody
**TBK1/NAK**	rabbit	Cell Signaling	D1B4	1:1000	Peroxidase AffiniPure $F(ab')_2$ Fragment Donkey Anti-Rabbit IgG (H+L)
**p-TBK1/NAK**	rabbit	Cell Signaling	D52C2	1:1000	Peroxidase AffiniPure $F(ab')_2$ Fragment Donkey Anti-Rabbit IgG (H+L)
**GAPDH**	mouse	GeneTex	1D4	1:1000	Peroxidase AffiniPure $F(ab')_2$ Fragment Donkey Anti-Mouse IgG (H+L)
**Actin**	rabbit	Sigma Aldrich	na	1:1000	Peroxidase AffiniPure $F(ab')_2$ Fragment Donkey Anti-Rabbit IgG (H+L)

Supplemental Table 6. Genes differentially represented in IFN-i^{Lo} and IFN-i^{Hi} AML patients (study cohort 1) as determined by RNA-sequencing.

Gene Symbol	Fold change (log2)	adj. p-value
COL14A1	4,55	0,0003
POU4F1	3,94	0,0001
ROBO1	3,41	0,0307
HCAR1	3,12	0,0022
MYO18B	2,78	0,0000
PCDHB13	2,54	0,0003
CSRP2	2,33	0,0018
PCDHB14	2,30	0,0017
CFAP47	2,23	0,0031
PPL	2,22	0,0017
PNMT	2,07	0,0015
FGF13	1,99	0,0046
PCDHGC3	1,98	0,0044
PCDHGA12	1,97	0,0042
KCNJ12	1,93	0,0110
PCDHGB6	1,91	0,0044
PCDHGB3	1,88	0,0010
SLC9A2	1,87	0,0093
PCDHGC5	1,82	0,0106
DLL3	1,81	0,0269
SVOPL	1,79	0,0273
PCDHGA1	1,78	0,0052
GPC6	1,78	0,0463
PSMA8	1,74	0,0423
PCDHGA2	1,73	0,0156
SELP	1,73	0,0204
PCDHGA3	1,71	0,0175
PCDHGC4	1,71	0,0157
LIMS2	1,69	0,0150
PCDHGB2	1,66	0,0353
AEBP1	1,65	0,0295
KLRF2	1,64	0,0372
REN	1,64	0,0292
ACKR2	1,64	0,0398
LTK	1,63	0,0172
IRX6	1,62	0,0108
PCDHGA6	1,58	0,0090
PCDHGA9	1,58	0,0267
APBA2	1,55	0,0185
PCDHGA7	1,49	0,0409

TRIM6	1,49	0,0305
APBA2	1,49	0,0244
PCDHGA8	1,48	0,0310
KCNH8	1,45	0,0494
TDRD9	1,44	0,0066
TMEM44	1,43	0,0066
ITPKA	1,43	0,0305
TMEM184A	1,42	0,0440
FBXL13	1,40	0,0240
SCN1B	1,37	0,0403
ADPRHL1	1,33	0,0134
NEK10	1,24	0,0227
DLGAP2	1,24	0,0260
TGFA	1,23	0,0461
RPS26	1,21	0,0007
NT5M	1,21	0,0110
TRH	1,16	0,0076
SCUBE3	1,13	0,0391
EPDR1	1,11	0,0192
SHD	1,02	0,0018
HSD17B1	1,01	0,0389
STMN3	0,98	0,0281
TSGA13	0,94	0,0377
FTO	0,82	0,0003
SLPI	0,80	0,0304
DDAH2	0,79	0,0075
LRWD1	0,78	0,0241
CCDC189	0,77	0,0106
PTPN7	0,75	0,0232
RAB37	0,74	0,0397
DDAH2	0,73	0,0156
TRIM24	0,72	0,0265
DDAH2	0,72	0,0162
DDAH2	0,72	0,0162
DDAH2	0,72	0,0162
MFRP	0,71	0,0198
DDAH2	0,64	0,0371
DDAH2	0,64	0,0371
SMYD5	0,61	0,0426
C1QTNF5	0,59	0,0167
CDAN1	0,57	0,0353
ESYT2	0,56	0,0293
DNAAF2	0,53	0,0157
SOCS2	0,34	0,0090
APBB2	0,30	0,0444
KCNIP3	0,29	0,0245

SLITRK5	0,28	0,0003
DEFB1	0,28	0,0397
KHDRBS3	0,28	0,0439
CDSN	0,27	0,0433
HS3ST1	-0,08	0,0178
CACNG4	-0,20	0,0061
ENHO	-0,27	0,0110
FAM120AOS	-0,33	0,0200
CSMD1	-0,34	0,0408
CLEC10A	-0,46	0,0114
NMNAT1	-0,56	0,0272
NAGS	-0,56	0,0350
RBMS3	-0,56	0,0427
FAM156A	-0,57	0,0377
NBPF20	-0,57	0,0447
FAM156B	-0,58	0,0370
TTC13	-0,60	0,0491
RAPGEF1	-0,60	0,0160
ZYG11B	-0,62	0,0230
AKIRIN2	-0,70	0,0371
ATP6V1A	-0,71	0,0369
SLCO2B1	-0,71	0,0211
SAMD8	-0,72	0,0420
SLC38A6	-0,75	0,0345
LRRC8C	-0,76	0,0197
C1QA	-0,79	0,0419
TRIM3	-0,80	0,0118
CLEC20A	-0,81	0,0059
ST18	-0,84	0,0042
ABR	-0,87	0,0466
ITPRIPL2	-0,87	0,0341
ZFAND5	-0,89	0,0308
GLTP	-0,90	0,0136
PLOD1	-0,90	0,0480
HOOK3	-0,92	0,0167
BMP2	-0,92	0,0000
TCF7L2	-0,92	0,0403
FKBP15	-0,94	0,0345
RPH3A	-0,95	0,0022
AC253572.1	-0,95	0,0389
SOAT1	-0,99	0,0397
ZSWIM6	-1,01	0,0494
PRKACA	-1,01	0,0235
PRKACA	-1,01	0,0235
CPPED1	-1,04	0,0452
SIDT2	-1,04	0,0213

CPNE3	-1,05	0,0348
RCBTB2	-1,06	0,0466
EVI5	-1,07	0,0270
CEMIP2	-1,07	0,0244
RAB24	-1,07	0,0397
SGTB	-1,09	0,0247
FTL	-1,09	0,0303
CHST2	-1,10	0,0422
FTH1	-1,11	0,0135
PLAUR	-1,12	0,0370
KIF1C	-1,13	0,0391
IFNGR2	-1,13	0,0247
IFNGR2	-1,14	0,0272
PLXNA1	-1,14	0,0453
SNTB2	-1,16	0,0136
OSBPL11	-1,16	0,0148
GAA	-1,17	0,0458
UBXN11	-1,18	0,0363
PLPPR2	-1,19	0,0039
SIMC1	-1,19	0,0125
HEXB	-1,19	0,0118
PILRA	-1,20	0,0277
PPM1N	-1,20	0,0340
RWDD2A	-1,21	0,0070
MILR1	-1,22	0,0399
TAGLN	-1,22	0,0110
ID2	-1,23	0,0203
C1orf54	-1,24	0,0403
RMDN2	-1,24	0,0180
ZFP36L1	-1,24	0,0466
IQSEC1	-1,25	0,0222
HEG1	-1,25	0,0374
MCUB	-1,25	0,0281
BCL2L11	-1,25	0,0175
AP1S2	-1,25	0,0180
IL4I1	-1,26	0,0094
PELI1	-1,28	0,0243
SLC35F6	-1,29	0,0041
HLA-B	-1,30	0,0397
SETBP1	-1,30	0,0381
TFEB	-1,30	0,0214
RIN1	-1,31	0,0221
NACC2	-1,35	0,0295
KCTD17	-1,35	0,0110
NAV2	-1,35	0,0447
NFKBIZ	-1,35	0,0260

PPEF2	-1,35	0,0122
TNFRSF8	-1,35	0,0322
ASPHD2	-1,35	0,0098
ARHGEF11	-1,35	0,0244
TBC1D8	-1,36	0,0071
PPFIA4	-1,36	0,0466
AC068580.4	-1,37	0,0099
ANKRD37	-1,37	0,0216
NIBAN2	-1,38	0,0482
BMERB1	-1,38	0,0478
CTSB	-1,38	0,0304
MEGF9	-1,38	0,0338
SLC31A1	-1,38	0,0044
SLC26A11	-1,40	0,0460
IFITM10	-1,41	0,0483
OTOA	-1,41	0,0225
POU2F2	-1,41	0,0241
PEAK3	-1,42	0,0292
CD93	-1,42	0,0481
RIN2	-1,42	0,0309
CMTM4	-1,43	0,0180
SLC18B1	-1,43	0,0066
AC007192.1	-1,44	0,0277
TNFAIP2	-1,46	0,0045
SNED1	-1,46	0,0185
SLC9A9	-1,46	0,0164
TGM2	-1,46	0,0423
SLC22A1	-1,46	0,0106
FGR	-1,48	0,0162
HORMAD1	-1,48	0,0114
NR4A1	-1,48	0,0449
VMO1	-1,49	0,0322
CDKL5	-1,49	0,0048
TNFRSF1B	-1,49	0,0208
SMCO4	-1,50	0,0094
PSAP	-1,51	0,0090
SCO2	-1,51	0,0334
MUC4	-1,51	0,0441
NOD2	-1,51	0,0402
MUC4	-1,51	0,0412
PLD3	-1,52	0,0058
SETD7	-1,52	0,0050
JAZF1	-1,52	0,0150
CHST15	-1,52	0,0452
AL049634.2	-1,52	0,0353
LRRC25	-1,53	0,0373

ZNF385A	-1,53	0,0134
CES4A	-1,54	0,0453
PLD2	-1,54	0,0166
CTSS	-1,54	0,0156
SLC43A2	-1,54	0,0048
MFSD2A	-1,54	0,0222
CTSD	-1,54	0,0050
AL355315.1	-1,55	0,0010
LILRB2	-1,56	0,0272
CACNB1	-1,56	0,0010
CD4	-1,56	0,0090
GALNT18	-1,56	0,0454
CPVL	-1,56	0,0345
SIRPB1	-1,57	0,0299
LILRA5	-1,57	0,0482
TICAM2	-1,58	0,0230
LILRA5	-1,58	0,0454
LILRA5	-1,58	0,0454
LILRA5	-1,58	0,0454
LILRA5	-1,58	0,0454
SLC43A2	-1,58	0,0037
TYMP	-1,58	0,0341
APOBEC3A	-1,58	0,0247
LILRB2	-1,58	0,0304
MARCKS	-1,59	0,0213
LILRA3	-1,59	0,0475
GRN	-1,60	0,0070
PCDH12	-1,60	0,0353
GABBR1	-1,60	0,0370
AC241409.1	-1,60	0,0170
IGF2R	-1,60	0,0010
RXRA	-1,60	0,0034
CXCL16	-1,62	0,0320
BAG3	-1,62	0,0074
RASSF4	-1,62	0,0059
ZNF703	-1,62	0,0347
FAM20A	-1,63	0,0006
TMEM170B	-1,63	0,0125
ADAP2	-1,63	0,0106
C9orf72	-1,63	0,0027
MS4A6E	-1,63	0,0226
LSAMP	-1,64	0,0094
CYB5R2	-1,64	0,0492
LILRA6	-1,64	0,0328
CYBB	-1,65	0,0263
RGL1	-1,65	0,0325

SCIMP	-1,65	0,0393
NINJ1	-1,66	0,0043
SGSH	-1,66	0,0110
ARHGAP29	-1,66	0,0474
LILRA1	-1,67	0,0096
FAM151B	-1,67	0,0049
NHS	-1,67	0,0389
TPRG1	-1,67	0,0340
CD14	-1,67	0,0481
GCNT2	-1,67	0,0015
GCNT2	-1,68	0,0014
PDE4A	-1,69	0,0088
C3	-1,69	0,0238
LILRB4	-1,69	0,0420
LYNX1	-1,69	0,0244
FOLR2	-1,70	0,0296
LILRA1	-1,71	0,0076
SLAMF8	-1,71	0,0062
LILRA1	-1,71	0,0072
LILRB4	-1,72	0,0397
SPON1	-1,72	0,0461
LILRA1	-1,72	0,0071
SGK1	-1,72	0,0157
PI4K2A	-1,72	0,0001
DNAJB5	-1,73	0,0045
SEPTIN10	-1,73	0,0277
LILRB5	-1,73	0,0241
HFE	-1,73	0,0169
SLC15A3	-1,74	0,0094
LILRB2	-1,74	0,0094
PHLDA2	-1,75	0,0157
PHLDA2	-1,75	0,0157
LILRB3	-1,75	0,0181
CAMK2D	-1,75	0,0007
TBC1D12	-1,76	0,0240
GASK1B	-1,76	0,0207
PPFIBP2	-1,76	0,0008
RUFY4	-1,76	0,0226
PTPRM	-1,77	0,0331
MMP17	-1,77	0,0166
CHIT1	-1,78	0,0272
IL15	-1,78	0,0205
COPZ2	-1,79	0,0162
LILRB2	-1,79	0,0059
CTSH	-1,79	0,0066
LIMCH1	-1,80	0,0322

MS4A14	-1,80	0,0070
LILRB5	-1,80	0,0157
SHROOM1	-1,81	0,0106
NWD1	-1,81	0,0331
CTSL	-1,81	0,0156
WNK2	-1,82	0,0022
CD300LB	-1,83	0,0097
SNX21	-1,83	0,0009
SLC37A2	-1,84	0,0150
LILRB2	-1,85	0,0043
CD1D	-1,85	0,0122
KLF4	-1,85	0,0075
S1PR3	-1,85	0,0188
PELI3	-1,85	0,0050
CHI3L1	-1,85	0,0240
IL19	-1,85	0,0122
HMOX1	-1,86	0,0101
VDR	-1,86	0,0027
PAPLN	-1,86	0,0136
SIRPD	-1,86	0,0121
PCSK5	-1,86	0,0024
ITGA7	-1,86	0,0039
NPL	-1,87	0,0051
CHRDL1	-1,87	0,0211
NMNAT2	-1,87	0,0267
CFAP99	-1,88	0,0199
EXT1	-1,89	0,0027
MS4A7	-1,89	0,0027
RGMA	-1,90	0,0178
LILRA6	-1,90	0,0104
MEIS3	-1,90	0,0185
CAMK1	-1,90	0,0135
GPBAR1	-1,90	0,0113
SHISA4	-1,91	0,0122
MT1H	-1,91	0,0086
CAVIN3	-1,91	0,0175
LGALS3	-1,92	0,0070
NLRP12	-1,92	0,0059
ACSM5	-1,92	0,0103
RAB11FIP5	-1,92	0,0044
CD300E	-1,93	0,0156
FGD5	-1,94	0,0045
SOWAHC	-1,94	0,0071
LILRB5	-1,95	0,0076
ACVR2A	-1,95	0,0001
LILRB5	-1,95	0,0076

FXYD6-FXYD2	-1,95	0,0022
CX3CR1	-1,96	0,0050
TSHZ3	-1,96	0,0024
LILRB5	-1,96	0,0073
LRP1	-1,97	0,0114
LILRB1	-2,00	0,0031
SDC3	-2,00	0,0094
AC113554.1	-2,03	0,0070
SLC16A2	-2,03	0,0094
LILRB1	-2,06	0,0020
CARD14	-2,07	0,0066
HMGA2	-2,07	0,0022
TMEM150B	-2,07	0,0027
MYCT1	-2,07	0,0050
CDKN2B	-2,07	0,0048
OR52K2	-2,08	0,0043
CCDC151	-2,08	0,0033
ASGR2	-2,08	0,0039
RAB39A	-2,08	0,0031
C8orf88	-2,10	0,0050
LILRB1	-2,10	0,0030
ISL2	-2,10	0,0051
LILRB1	-2,11	0,0018
SERINC2	-2,11	0,0027
FCN2	-2,12	0,0034
LILRB1	-2,12	0,0020
TWIST2	-2,14	0,0037
TWIST2	-2,14	0,0037
CHST1	-2,15	0,0053
MYO7A	-2,15	0,0048
CLEC4G	-2,17	0,0045
PDK4	-2,17	0,0034
KLK1	-2,17	0,0018
MAFB	-2,18	0,0037
CADM1	-2,18	0,0017
VCAN	-2,20	0,0039
ACE	-2,22	0,0017
PLAAT3	-2,24	0,0003
PKD2L1	-2,25	0,0005
HTR7	-2,27	0,0010
MT1E	-2,29	0,0010
RAPH1	-2,29	0,0007
CYP27A1	-2,32	0,0007
TMEM176B	-2,33	0,0017
RGL3	-2,35	0,0016
FXYD6	-2,36	0,0003

IL31RA	-2,37	0,0005
TRIM7	-2,38	0,0003
CES1	-2,40	0,0009
CACNB2	-2,44	0,0118
TMEM176A	-2,45	0,0007
SLC8A1	-2,46	0,0001
CES1	-2,46	0,0006
PID1	-2,49	0,0004
ZNF462	-2,50	0,0198
SDC2	-2,53	0,0004
EPB41L3	-2,53	0,0003
TNNT1	-2,54	0,0003
KNDC1	-2,59	0,0002
CDC42EP1	-2,60	0,0002
SLC46A2	-2,61	0,0002
KAZALD1	-2,64	0,0002
MLXIPL	-2,64	0,0227
ASGR1	-2,66	0,0000
FCN1	-2,66	0,0001
PLA2G2C	-2,73	0,0001
SMPDL3A	-2,74	0,0001
CYP1B1	-2,77	0,0001
TRPV4	-2,78	0,0001
CACNG8	-2,84	0,0094
SASH1	-2,87	0,0000
